# HSV-2 Infection of Dendritic Cells Amplifies a Highly Susceptible HIV-1 Cell Target

**DOI:** 10.1371/journal.ppat.1002109

**Published:** 2011-06-30

**Authors:** Elena Martinelli, Hugo Tharinger, Ines Frank, James Arthos, Michael Piatak, Jeffrey D. Lifson, James Blanchard, Agegnehu Gettie, Melissa Robbiani

**Affiliations:** 1 Center for Biomedical Research, Population Council, New York, New York, United States of America; 2 Laboratory of Immunoregulation, National Institute of Allergy and Infectious Diseases, National Institutes of Health, Bethesda, Maryland, United States of America; 3 AIDS and Cancer Virus Program, SAIC-Frederick, Inc., National Cancer Institute, Frederick, Maryland, Unites States of America; 4 Tulane National Primate Research Center, Tulane University Health Sciences Center, Covington, Louisiana, United States of America; 5 Aaron Diamond AIDS Research Center, Rockefeller University, New York, New York, Unites States of America; University of Pennsylvania School of Medicine, United States of America

## Abstract

Herpes simplex virus type 2 (HSV-2) increases the risk of HIV-1 infection and, although several reports describe the interaction between these two viruses, the exact mechanism for this increased susceptibility remains unclear. Dendritic cells (DCs) at the site of entry of HSV-2 and HIV-1 contribute to viral spread in the mucosa. Specialized DCs present in the gut-associated lymphoid tissues produce retinoic acid (RA), an important immunomodulator, able to influence HIV-1 replication and a key mediator of integrin α_4_β_7_ on lymphocytes. α_4_β_7_ can be engaged by HIV-1 on the cell-surface and CD4^+^ T cells expressing high levels of this integrin (α_4_β_7_^high^) are particularly susceptible to HIV-1 infection. Herein we provide *in-vivo* data in macaques showing an increased percentage of α_4_β_7_^high^ CD4^+^ T cells in rectal mucosa, iliac lymph nodes and blood within 6 days of rectal exposure to live (n = 11), but not UV-treated (n = 8), HSV-2. We found that CD11c^+^ DCs are a major target of HSV-2 infection in *in-vitro* exposed PBMCs. We determined that immature monocyte-derived DCs (moDCs) express aldehyde dehydrogenase ALDH1A1, an enzyme essential for RA production, which increases upon HSV-2 infection. Moreover, HSV-2-infected moDCs significantly increase α_4_β_7_ expression on CD4^+^ T lymphocytes and HIV-1 infection in DC-T cell mixtures in a RA-dependent manner. Thus, we propose that HSV-2 modulates its microenviroment, influencing DC function, increasing RA production capability and amplifying a α_4_β_7_^high^CD4^+^ T cells. These factors may play a role in increasing the susceptibility to HIV-1.

## Author Summary

The vast majority of HIV-1 infections occur through genital and rectal mucosa. A better understanding of the characteristics of the mucosal microenvironment that help HIV-1 replication is critical to developing strategies for prevention of HIV-1 transmission. HSV-2 infects genital and rectal mucosa and infected individuals carry an increased risk for HIV-1 infection. Clarifying the mechanisms involved in the increased susceptibility of HSV-2 positive individuals to HIV-1 infection may help understating the characteristics of mucosal microenvironment that facilitate HIV-1 transmission. We previously described a specific interaction between HIV-1 and integrin α_4_β_7_, a signature molecule that allows lymphocytes to gain access to the gut tissue, a major site of HIV-1 replication. Vitamin A and its metabolite, retinoic acid, have an important role in balancing the immune response in the gut and in the expression of integrin α_4_β_7_. Here we describe that HSV-2 rectal infection in monkeys increases the frequency of α_4_β_7_^+^ CD4^+^ T cells in blood and rectal tissue and that this could be at least partially explained by the ability of HSV-2 infected DCs to secrete retinoic acid and up-regulate α_4_β_7_ on CD4^+^ T cells. These phenomena could be responsible for increasing HIV-1 replication in DC-T cell co-cultures.

## Introduction

Herpes Simplex Virus Type 2 (HSV-2) infects genital and perianal mucosa and its infection is associated with a three-fold increased risk of HIV-1 acquisition among both men and women [1]. Although active HSV-2 shedding, inflammation and ulcers during primary infections and virus reactivation certainly contribute, their resolution by suppressive therapy with acyclovir is not effective in reducing HIV-1 acquisition in HSV-2 seropositive individuals [2]. One possible explanation for the HSV-2-driven increased risk of HIV-1 acquisition is the persistence of HSV-2-reactive CD4^+^ T cells long after HSV-2 replication abates [3]. Likewise, plasmacytoid and myeloid dendritic cells (DCs), which infiltrate areas of skin infected with HSV-2, persist after lesion healing also in the context of acyclovir therapy [3] and may contribute to the increased risk of HIV-1 acquisition associated with HSV-2 infection.

Epithelial cells are primary targets of HSV-2 infection. Nonetheless, DCs, which orchestrate the immunological response to HSV-2 at its portal of entry, can also be infected *in-vitro*. In fact immature monocyte-derived DCs (moDCs) and langerhans cells found at the epithelial surfaces are highly susceptible to HSV-2 [4], [5], [6], [7]. Importantly, HSV-2 infection of DCs *in-vitro* has been shown to inhibit their maturation and immunostimulatory functions [5], [6], [8], [9] and *in-vivo* HSV-2 infection reduces HIV-1 specific T cell responses [6], [10], [11].

Cellular microenvironment is vital to conditioning cell function and, in particular, the expression of receptors that affect cell trafficking. Specialized DCs in mesenteric lymph nodes (MLNs) and Peyer's patches (PPs) convert vitamin A to retinoic acid (RA) [12], a key factor in the control of lymphocyte trafficking and immune responses and able to influence HIV-1 replication [13], [14], [15]. In particular, RA has the unique capacity to imprint a “gut-phenotype” on T cells, which includes increased expression of integrin α_4_β_7_ [12]. The mucosal homing receptor α_4_β_7_ is the signature molecule that allows lymphocytes to gain access to the gut tissue [16], [17], a major site of HIV-1 replication [18]. A recent study in macaques has shown that pre-treatment with an anti-α_4_β_7_ antibody significantly reduces and delays peak plasma SIV load, increases the percentage of CD4^+^ T cells both in peripheral blood and in gut tissues and reduces proviral DNA in blood and gut tissues mononuclear cells [19]. A specific interaction between α_4_β_7_ and the HIV-1 envelope protein, gp120, has been described [20] and additional evidence shows that α_4_β_7_ co-localizes with the two main HIV-1 entry receptors CD4 and CCR5 [21], [22]. The site of gp120 binding to α_4_β_7_ is conserved across gp120s from the four major HIV-1 subtypes [20]. The conserved nature of this interaction suggests that engaging α_4_β_7_ provides a selective advantage to HIV-1. Indeed, α_4_β_7_ is expressed at high levels on a subset of CD4^+^ T cells that are particularly susceptible to infection and it has been proposed that the specific affinity of HIV-1 gp120 for α_4_β_7_ provides a way for HIV-1 to target susceptible cells at an early stage of transmission [21]. Notably, very high reactivity for α_4_β_7_ is a characteristic shared by early transmitted isolates, in contrast with the poor reactivity of viruses isolated longer after transmission [23]. Once HIV-1 crosses the epithelium, they have to replicate at a reproductive rate R_0_>1 [24]. Expansion of the virus within the mucosa is necessary to disseminate infection to draining LNs and blood in order to establish a productive systemic infection. The relevance of the HIV-α_4_β_7_ interaction in the context of mucosal transmission has yet to be elucidated.

The synthesis of RA includes two main steps. During the first step vitamin A (retinol) is converted to retinaldehyde. This reaction is catalyzed by a subfamily of alcohol dehydrogenases that are expressed in most cells including DCs or by the short-chain dehydrogenase/reductase family [12], [25], [26]. The second, limiting step, is catalyzed specifically by aldehyde dehydrogenase 1A (ALDH1A) [retinal dehydrogenase] which converts retinaldehyde to RA. ALDH1A-expression *in-vivo* does not appear to be restricted to intestinal DCs and intestinal epithelial cells as previously suggested [25], [27], [28]. ALDH1A can be expressed by LN stromal cells [29], [30] and ALDH1A expressing-RA-producing DCs are present in skin, lung and in their draining LNs [31]. Moreover, RA production can be induced *in-vitro* in splenic and bone marrow-derived DCs (BM-DCs) [32]. In this regard, the granulocyte-macrophage colony-stimulating factor (GM-CSF) and stimulation with toll-like receptor (TLR) ligands appear to play an important role [32], [33]. This raises the possibility that ALDH1A can be expressed and induced in DCs in various tissues outside of the intestine.

The present work shows that rectal HSV-2 infection of macaques increases the percentage of α_4_β_7_^high^CD4^+^ T cells in rectal tissue and draining LNs, as well as systemically. We found that immature moDCs express ALDH1A and this is upregulated by HSV-2 infection. Therefore, HSV-2 increases DC's ability to produce RA. In turn HSV-2 infected moDCs, induce α_4_β_7_ expression expanding the α_4_β_7_^high^ CD4^+^ T subset through an RA-dependent mechanism. Importantly, HSV-2 infected moDCs increase HIV-1 replication in DC-T cell co-cultures in an RA-dependent manner. Herein, we describe a mechanism that HSV-2 could exploit to condition its environment, influencing not only its own replication but, possibly, that of a co-pathogen such as HIV-1.

## Results

### Rectal HSV-2 infection of macaques increases the percentage of α4β7^high^ T cells

Non-human primate models constitute crucial tools to explore mucosal infection with HIV-1/SIV, elucidate the early events after HIV-1 transmission and how these events are impacted by other sexually transmitted infections (STIs). We recently developed a unique model of vaginal HSV-2 infection in rhesus macaques in which HSV-2-infected macaques exhibited increased susceptibility to immunodeficiency virus (simian-human immunodeficiency virus, SHIV-RT) infection, even in absence of obvious lesions [10]. Building on this, we established a macaque model of HSV-2 rectal infection and we used this model to investigate early events after HSV-2 infection across the mucosa. Clinically stable SHIV-RT-infected animals were challenged intra-rectally with 2×10^8^ pfu of replication competent (LIVE; n = 11) or UV-inactivated (UV-HSV-2; n = 8) HSV-2. UV inactivated HSV-2 (matched to the input infectious virus dose prior to inactivation) was used to distinguish effects due to exposure to viral proteins and nucleic acids from the effects due to viral replication. Baseline CD4 counts and plasma SHIV RNA levels are listed for each animal in (Table S1). Blood and rectal fluids were collected before (BL), 3 and 6 days after challenge. Axillary, mesenteric, inguinal and iliac LNs, as well as rectal tissue were collected following euthanization 6 days after challenge.

HSV-2 DNA levels were measured in the rectal fluids at BL (4 days and 24 h prior to HSV-2 challenge. 24 h is shown in Table S1) versus 3 and 6 days post-challenge by a sensitive nested HSV-2 PCR [10]. HSV-2 shedding was detected in 9 of the 11 animals challenged with live HSV-2 on days 3 and 6 post-challenge (Table S1). One animal was negative on day 6 and unable to be tested on day 3 and one was negative on day 3 and unable to be tested at day 6. As a result, these animals were excluded from subsequent analyses since their HSV-2 status was unclear. No DNA shedding was detected in the animals treated with UV-HSV-2, nor in any animals prior to challenge. Not surprisingly, CD4 counts and plasma SHIV RNA did not show significant changes from baseline 6 days after exposure to live or UV-inactivated HSV-2 (Table S1).

**Figure 1:**
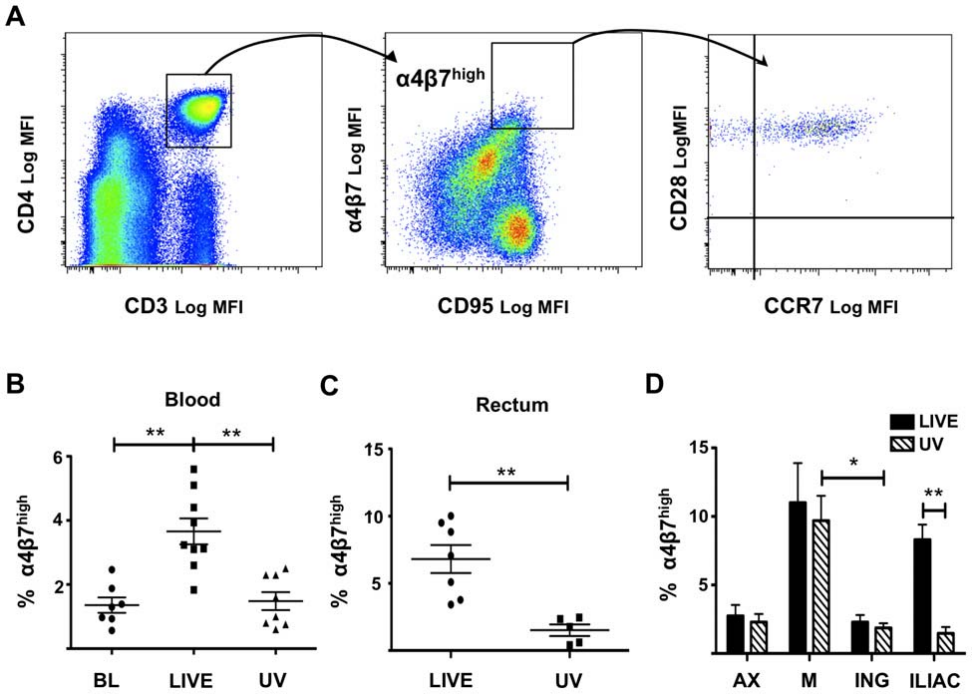
Rectal HSV-2 challenge increases the percentage of α_4_β_7_^high^CD3^+^CD4^+^ T cells in *in-vivo*. A) The gating strategy for α_4_β_7_^high^CD3^+^CD4^+^CD95^+^ T cells in blood is shown from one representative animal. The majority of the α_4_β_7_^high^ T cells are CD95^+^CD28^+^CCR7^+^ (last plot on the right). B) The percentages (mean ± SEM) of CD3^+^CD4^+^CD95^+^ T cells that are α_4_β_7_^high^ in blood are shown at 4 days before infection (BL) and at 6 days p.i. for HSV-2-infected (LIVE, n = 9) and UV-HSV-2-treated (UV, n = 8) animals. C) The percentages (mean ± SEM) of CD3^+^CD4^+^ T cells that are α_4_β_7_^high^ in rectal mucosa are shown for HSV-2-infected (LIVE, n = 7) and UV-HSV-2-treated (UV, n = 5) animals 6 days p.i.. D) The percentages (mean ± SEM) of CD3^+^CD4^+^CD95^+^ T cells that are α_4_β_7_^high^ in axillary (AX), mesenteric (M), inguinal (ING), and iliac LNs are shown for HSV-2-infected (LIVE, n = 5) and UV-HSV-2-treated (UV, n = 7) animals. B–C) Each symbol represents an animal. (*p<0.05, **<0.01, ***p≤0.001).

Previous studies have shown that the α_4_β_7_^high^CD4^+^ T cells constitute a subset of central memory cells [21], [22] and this was similarly observed in the present study (Figure 1A). In blood of macaques infected intra-rectally with HSV-2 we detected a significantly higher percentage of α_4_β_7_^high^ T cells within the CD4^+^CD95^+^ memory cell subset than before challenge and than in macaques challenged with UV-HSV-2 (Figure 1B). The frequency of CD4^+^CD95^+^α_4_β_7_^high^ T cells was also higher in the rectal mucosa of the HSV-2 infected macaques than in the UV-HSV-2 treated ones. (Figure 1C; S1 shows the gating strategy). The percentage of α_4_β_7_^high^ cells in the CD4^+^ T cell memory subset was also significantly increased in the draining iliac LNs of HSV-2 infected macaques (Figure 1D). As expected, the gut-draining MLNs had the highest percentage of α_4_β_7_^high^CD4^+^ T cells and, together with the low levels in the distal axillary LNs, they were unaffected by HSV-2 infection (Figure 1D). Of note, the inguinal LNs of infected macaques also had a higher frequency of α_4_β_7_^high^ cells than the UV-HSV-2 treated animals. Although this difference was not significant, finding that inguinal LNs of UV-HSV-2-treated macaques had a significantly lower percentage of α_4_β_7_^high^ cells than the MLNs while this different was not significant in the live-HSV-2 treated ones, suggests that inguinal LNs of the HSV-2 infected macaques are also enriched in α_4_β_7_^high^CD4^+^ T cells (Figure 1D). All together, these data suggest that HSV-2 infection is either recruiting α_4_β_7_^high^ T cells to the infected mucosa and in draining LNs, or it is driving an up-regulation of α_4_β_7_ on T cells resident at the site of infection.

### Low-level HSV-2 infection modulates moDC function

**Figure 2:**
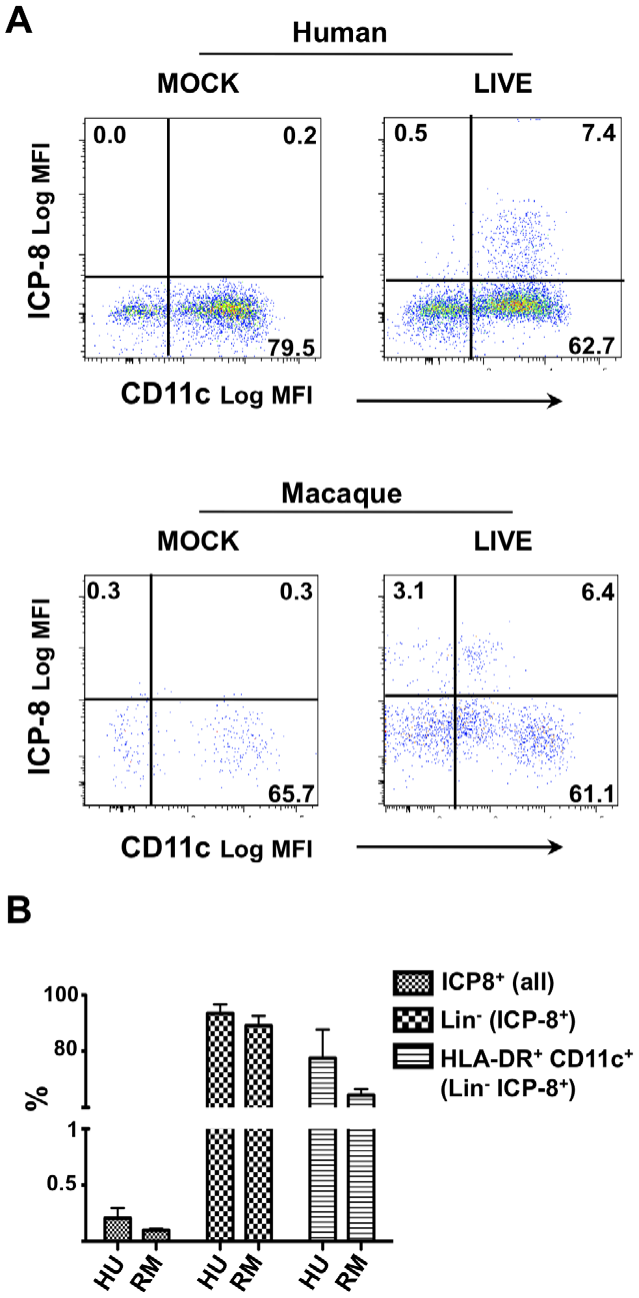
Blood CD11c^+^ DCs are susceptible to HSV-2 infection. A) Human (n = 3) and macaque (n = 3) PBMCs were HSV-2-infected (5 MOI, LIVE) or mock-treated and the ICP-8 expression measured 24 h later. ICP-8 versus CD11c expression is shown for Lin^−^HLA-DR^+^ cells on representative examples. The percentages of the positively stained cells are provided. B) The percentages (mean ± SEM of 3 donors each) of total live PBMCs that are ICP8^+^, of ICP8^+^ cells that are Lin^−^, and of the ICP8^+^Lin^−^ cells that are HLA-DR^+^CD11c^+^ are shown for human (HU) and macaque (RM) cultures.

In mucosal tissues α_4_β_7_ is up-regulated on T cells by RA produced by local DCs, therefore we set out to explore the possible involvement of DCs in the HSV-2-driven up-regulation of α_4_β_7_ in the rectally challenged macaques. While epithelial cells are the primary targets of HSV-2 infection, human and macaque moDCs are susceptible to HSV-2 *in-vitro* [4], [5], [6], [7]. Rationalizing that HSV-2 is interacting with a mixed population of leukocytes *in-vivo*, we used peripheral blood cells to examine which cells become infected by HSV-2 upon exposure *in-vitro*. Using a flow cytometric assay able to detect the HSV-2 early DNA binding protein ICP-8 intracellularly in infected cells, we determined that 24 h after exposure to HSV-2 (5 MOI), on average, 0.15% [range 0.08–0.39%] of total live cells were ICP-8^+^, 91.3% [range 82–99%] of the ICP-8^+^ were Lineage^−^ (Lin^−^) and 71% [range 62–95%] of the Lin^−^ICP-8^+^ were HLA-DR^+^CD11c^+^ (Figure 2B). Cell viability was always in the range 90–95% in human PBMCs and 50–80% in macaques and was not affected by HSV-2 infection. Although fewer, ICP-8^+^CD11c^+^ DCs were detected also after exposure to only 0.2 MOI of HSV-2 (not shown). Therefore, CD11c^+^ myeloid DCs were found to be the cell subset most susceptible to HSV-2 infection in human and macaque peripheral blood mixed leukocyte populations, supporting the possibility that HSV-2 infection of DCs plays a role *in-vivo*.

**Figure 3:**
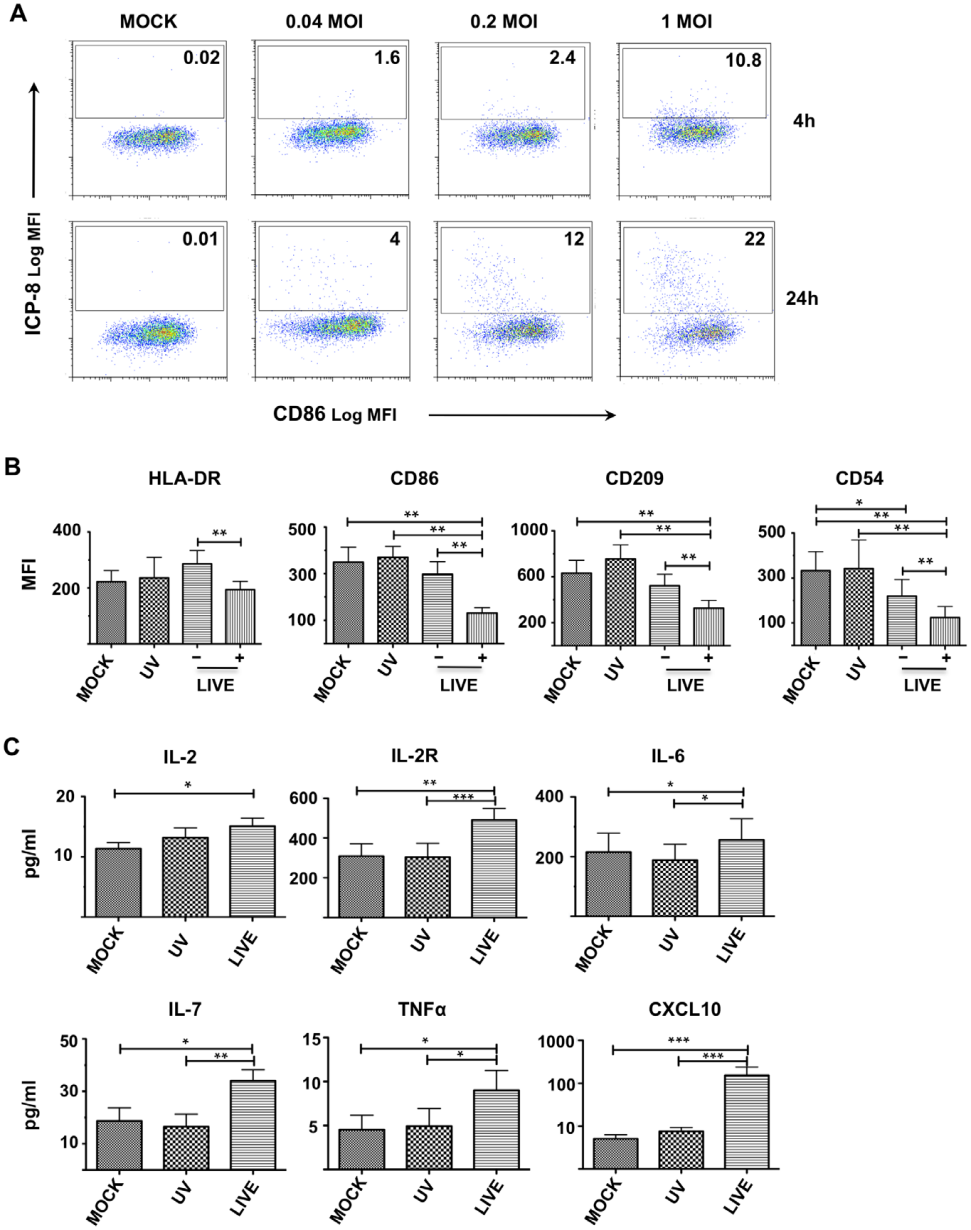
Low level HSV-2 infection modulates DC function. A) Immature moDCs were exposed to 0.04, 0.2, or 1 MOI of replication competent HSV-2 (or medium, MOCK) and infection was monitored by measuring the expression of ICP-8 at 4 h and 24 h. The percentage of ICP-8^+^ (infected) cells is indicated in each panel. One representative experiment of 5 is shown. B) DCs were treated with 0.2 MOI of replication competent (LIVE) or UV-HSV-2 (UV) versus mock (MOCK) supernatant and the surface phenotype assessed after 24 h. The mean fluorescent intensities (MFI) (means ± SEM, 5 independent experiments) of the indicated markers are shown for the total mock (MOCK) and total UV-HSV-2-treated (UV) DCs versus the ICP-8^−^ (−) and ICP-8^+^ (+) fractions of the LIVE condition. C) CK/CC concentrations (means ± SEM, 11 independent experiments) in the cell-free supernatants from LIVE-, UV-, and MOCK-treated moDCs are shown. (*p<0.05, **<0.01, ***p≤0.001).

In order to dissect this biology more extensively, we utilized the moDC system. Prior studies demonstrated that moDCs infected with HSV-2 at a MOI of 5 undergo rapid apoptosis [6], [34]. In order to explore the possibility that HSV-2 infection of DCs is involved in the up-regulation of α_4_β_7_ on CD4^+^ T on cells, we evaluated the effect of a low-level (minimally toxic) HSV-2 infection of DCs and how this influences DC-T cell interplay. We monitored infection of immature moDCs exposed to varying doses of HSV-2 by ICP-8 intracellular staining and we observed a dose-dependent infection that increased over time (Figure 3A). Annexin V/propidioum Iodide (PI) and the LIVE/DEAD discriminator marker Aqua were used to determine cell viability [35]. The percentage of Annexin V^−^ cells was similar to the percentage of Aqua negative cells, which represents our viable cell population. Therefore, we chose to use the LIVE/DEAD Aqua throughout the following studies. On average 80% [range 76–85%] and 55% [range 44–58%] of the moDCs were viable after infection with 0.2 and 1 MOI of HSV-2 (respectively), but there was little/no death of cells infected with 0.04 MOI (Figure S2). From these data we determined that the 0.2 MOI inoculum was lowest amount of virus giving reliable infection while leaving the majority of cells healthy and able to interact with T cells for our DC-T cells experiments.

Infections with higher doses of HSV-2 have been shown to have immunosuppressive effects on DCs [5], [6]. Therefore, changes in DC surface phenotype and cytokine/chemokine (CK/CC) profiles were monitored after HSV-2 infection with this lower inoculum. There were no significant differences in the expression of the surface markers and in the concentration of the CCs/CKs released by moDCs 4 h after exposure to infectious (live) or UV-HSV-2 (versus mock infected DCs; data not shown). Although the expression of CD80, CD83, CD40 and CD25 were comparable between the differently treated groups (data not shown), the expression of HLA-DR, CD86, CD209 and CD54 were all significantly reduced 24 h after HSV-2 infection (Figure 3B). HLA-DR was significantly lower in the ICP-8^+^ (infected) fraction of the cells exposed to infectious HSV-2, compared to the ICP-8^−^ (undetectable uninfected or low-level infected) subset. CD86, CD209, and CD54 were significantly reduced in the ICP-8^+^ cells compared to ICP-8^−^ cells within the infected population, as well as compared to the UV-HSV-2 and mock-treated controls.

Low level HSV-2 infection of human moDCs did not alter the release of IL1β, IL1RA, IL-5, CXCL8, IL-10, IL-12p40, IL-13, IL-15, IL-17, IFNα, IFNγ, CCL2, CCL3, CCL4, CXCL9, CCL11, CCL5 (data not shown). However, the levels of IL-2, IL-2R, IL-6, IL-7, TNFα, and CXCL10 were significantly increased in the HSV-2 infected DC cultures (Figure 3C). Notably, the amount of CXCL10 in the supernatants of HSV-2 infected DCs was, on average, 25 fold [95%CI 5–45] higher than both UV-HSV-2 and mock-infected cells. Overall, these results indicate that the infection of moDCs with 0.2 MOI of HSV-2 minimizes apoptosis in the first 24 h post infection (p.i.), retaining the ability of HSV-2 infection to modulate DC phenotype and function. This allowed us to study how the HSV-2 driven changes on DCs influence the DC-T interplay.

### HSV-2 infected moDCs induce α_4_β_7_ on CD4^+^ T cells via RA

**Figure 4:**
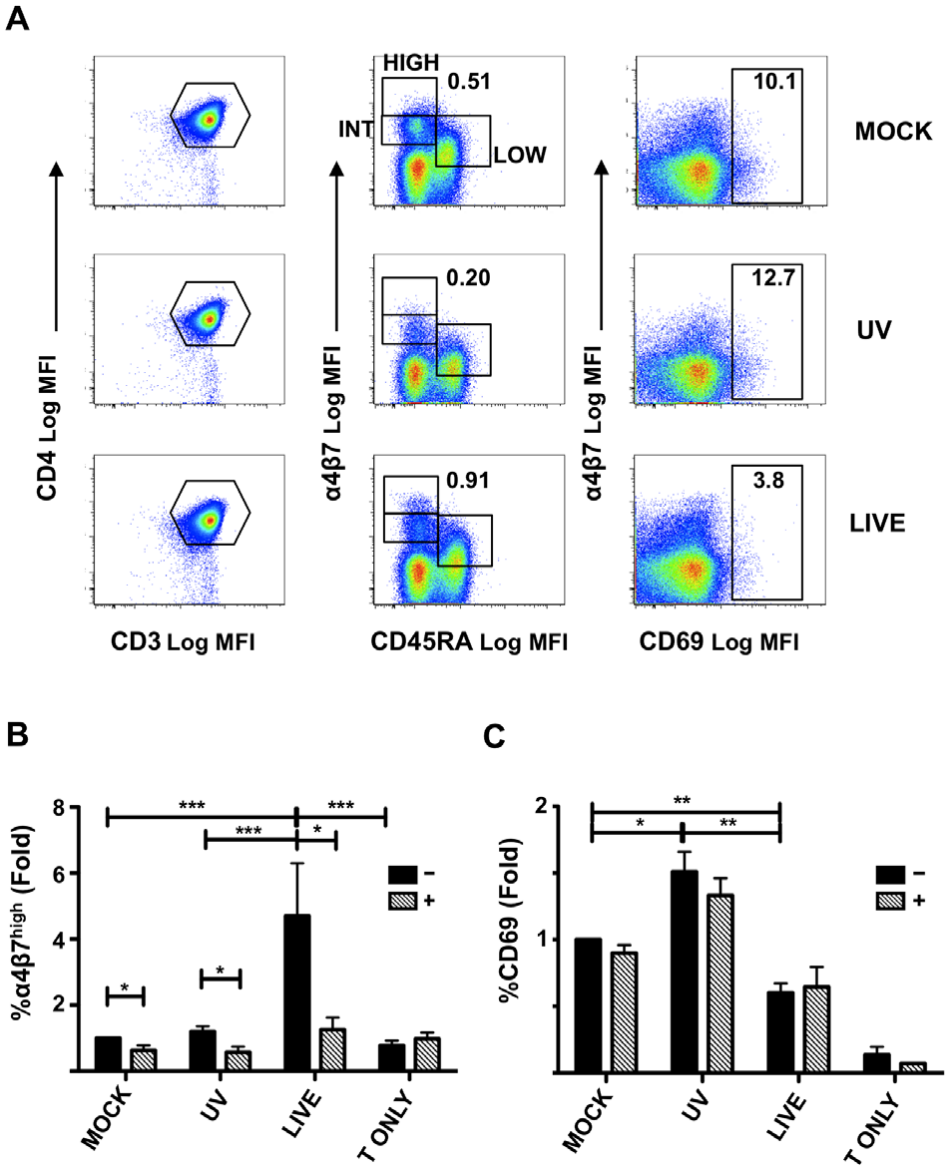
HSV-2 infection of moDCs induces α_4_β_7_ on CD4^+^ T cells. Mock-, UV-HSV-2-, or Live HSV-2-treated DCs (Figure 3) were mixed with autologous CD4^+^ T cells and cultured for 5 days. A) The gating strategy for the definition of the α_4_β_7_^high^ (HIGH) (memory cells), α_4_β_7_^int^ (INT) (memory cells) and α_4_β_7_^low^ (LOW) (naïve cells) and CD69^+^ subsets is shown for 1 representative of 11 independent experiments. B–C) The fold changes (mean ± SEM, 11 independent experiments) in the percentage of α_4_β_7_^high^CD3^+^CD4^+^ T cells (B) and CD69^+^CD3^+^CD4^+^ T cells (C) in the absence (−) or presence (+) of an RARα antagonist are shown relative to the mock-treated controls (set as 1). A T cell only control (no DCs, T ONLY), not exposed to HSV-2, was included alongside. (*p<0.05, **<0.01, ***p≤0.001).

CD4^+^ T cells cultured for 5 days with autologous DCs can be divided in 4 subsets on the basis of their expression of α_4_β_7_. While α_4_β_7_^low^ T cells (LOW) are naïve T cells, α_4_β_7_^int^ (INT), α_4_β_7_^high^ (HIGH) and α_4_β_7_^neg^ present a memory phenotype (Figure 4A and S3). After 5 (and 7, not shown) days of culture, the percentage of α_4_β_7_^high^ T cells was significantly increased in HSV-2-infected DC-T cell co-cultures (Figure 4A and B), while there was no significant difference in the α_4_β_7_^int^ and α_4_β_7_^low^ (not shown). In contrast, CD69 expression was significantly reduced in co-cultures with HSV-2-infected DCs, (relative to the mock and UV-treated cells; Figure 4A and C). It has been shown that the expression of α_4_β_7_ on naïve T cells is enhanced upon antigenic stimulation with MLN-DCs and PP-DCs [12]. Therefore, the small, non-significant increase in α_4_β_7_ expression seen in the UV-HSV-2 condition could be explained by stimulation of TLRs due to HSV-2 proteins and nucleic acids, the amount of which would be increased in cultures with replicating virus. However, even treatment of moDCs with 25 fold more UV-HSV-2 (equivalent to an MOI of 5), did not significantly alter the α_4_β_7_ expression on the CD4^+^ T cells, but increased CD69 expression even further (Figure S4 A and B).

T cells cultured alongside in the absence of DCs, but in the same amounts of live-HSV-2 used to treat the DCs (0.2 MOI), did not show evidence of infection (Figure S5 A), alteration of viability status (not shown) or changes in the expression of α_4_β_7_ (Figure S5 B) 5 days p.i. However, as previously reported [36], T cells treated with a higher inoculum are susceptible to HSV-2 infection, as demonstrated by the detection of ICP-8^+^CD4^+^ T cells after exposure to 5 MOI of HSV-2 (Figure S5 A). T cell viability and expression of α_4_β_7_ remained unchanged after exposure to the higher dose of HSV-2 (Figure S5 B). On the contrary, expression of CD69 was significantly up-regulated on the T cells 5 days post HSV-2 infection both in cells infected with 0.2 and 5 MOI (Figure S5 C).

Since RA is known to increase α_4_β_7_ expression in T cells, a selective RA receptor (RARα) antagonist [37] was included in the co-cultures. The RARα antagonist blocked the HSV-2-infected DC-induced up-regulation of α_4_β_7_ on the T cells (Figure 4B). The slight increase in α_4_β_7_^high^ cells in the UV-HSV-2-treated compared to mock-infected DC-T cell co-cultures was also blocked (Figure 4B). The RARα antagonist reduced α_4_β_7_ also on the mock-infected DC-T co-cultures, although its effect was much less pronounced than in the HSV-2 infected and UV-HSV-2 DC-T cell co-cultures (Figure 4B). Of note, CD4^+^ T cells cultured for 5 days in absence of moDCs, but in the same culturing conditions as the DC-T cell co-cultures, expressed a lower level of α_4_β_7_ than in presence of mock, UV-HSV-2 and HSV-2 moDCs and the antagonist had no effect on its expression (Figure 4B). The RARα antagonist did not alter the CD69 expression (Figure 4C), suggesting that its effect was highly specific, did not generically alter the viability status of the cells, and that RA is not involved in the reduced CD69 expression by the T cells. These data demonstrate that RA is at least partially responsible for inducing expression of α_4_β_7_ on the CD4^+^ T cells.

### HSV-2 infection increases ALDH1A1 expression in moDCs

The irreversible enzymatic conversion of retinaldehyde to *all-trans*-RA by ALDH1A [RALDH] constitutes the limiting step in the production of metabolically active RA. There are 3 main isoforms of ALDH1A. ALDH1A1 [RALDH1] is expressed by DCs in PPs and by epithelial cells of the intestine, while ALDH1A2 [RALDH2] is expressed in MLNs [12]. ALDH1A3 is expressed at much lower levels in both PPs and MLNs. Peripheral LNs express ALDH1A2 but at a barely detectable level [12]. Murine BM-DCs, differentiated in GM-CSF and IL-4, express ALDH1A2 [32]. We investigated whether human moDCs express ALDH1A and if the expression of α_4_β_7_ on T cells in the DC-T cell co-cultures could be related to a *de-novo* production of RA by the DCs.

**Figure 5:**
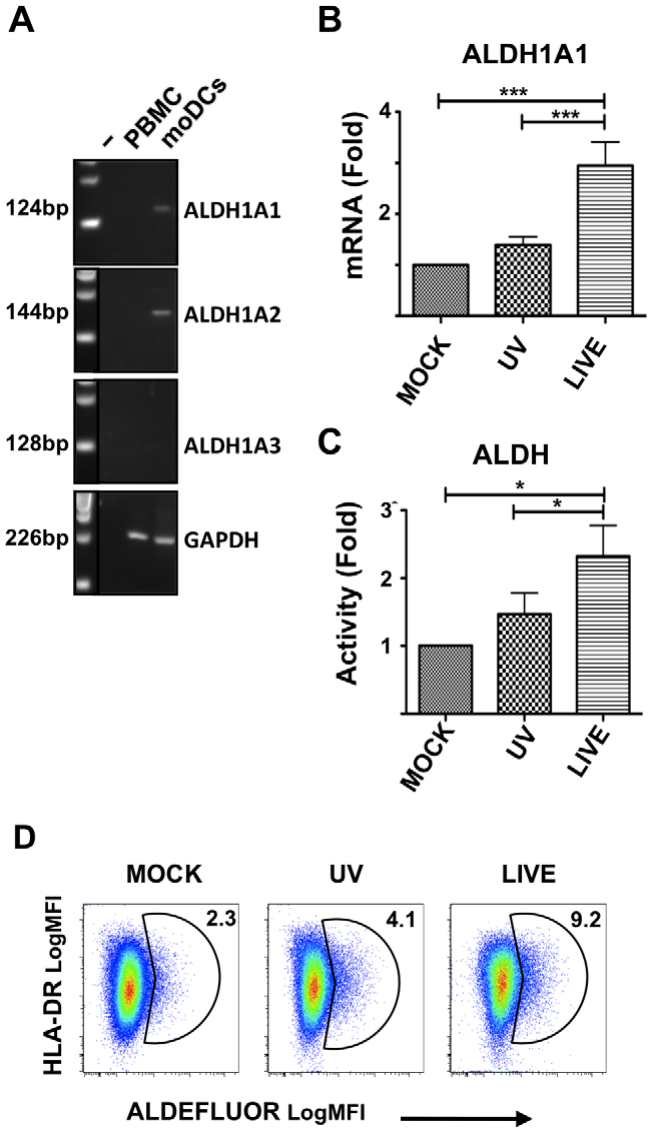
HSV-2 infection increases ALDH1A1 expression in moDCs. A) Expression of ALDH1A1, ALDH1A2, and ALDH1A3 RNA in immature moDCs and PBMCs by RT-PCR. Results shown are representative of data from 2 different donors. A negative control (−) without DNA was included to control for contamination. GAPDH was amplified in each sample as an internal PCR and loading control. B) The fold changes (mean ± SEM, 13 independent experiments) in ALDH1A1 mRNA of LIVE- and UV-HSV-2-treated moDCs (24 h post treatment) are shown relative to mock-treated moDCs (set as 1). C) The fold changes (mean ± SEM, 5 independent experiments) in the percentage of ALDEFLUOR positive (ALDH enzymatic activity) moDCs treated with LIVE or UV-HSV-2 (24 h post treatment) are shown relative to mock-treated moDCs (set as 1). D) Gating strategy for ALDH enzymatic activity in mock-, UV-HSV-2-, or Live HSV-2-treated moDCs (summarized in panel C). One representative of 5 independent experiments is shown. The numbers indicate the percentage of ALDH^+^ cells. (*p<0.05, **<0.01, ***p≤0.001).

We found that immature moDCs express ALDH1A1 and ALDH1A2, but not ALDH1A3 (Figure 5A). A relative quantification of the expression levels by RT-qPCR indicated that HSV-2 infection significantly increased the expression of ALDH1A1 compared to the UV-HSV-2- and mock-treated controls (Figure 5B). It has been shown that TLR ligands enhance ALDH1A2 expression in BM-DCs [32]. However, although the UV-HSV-2-treated moDCs expressed higher level of ALDH1A1 compared with the mock-treated cells, this difference was not significant. Moreover, the treatment with 25-fold more UV-HSV-2 did not significantly up-regulate ALDH1A1 in immature moDCs (Figure S6 A). We also found that generic aldehyde dehydrogenase activity (ALDH) increases significantly in HSV-2-infected moDCs compared with the UV-HSV-2 treated and mock-treated controls (Figure 5C and D). Because the assay measuring ALDH activity could not be used in combination with ICP-8 intracellular staining we could not distinguish if the increased ALDH activity was restricted to the HSV-2 infected cells, or if there was contribution from bystander non-infected cells. Thus, these data indicate that human moDCs have the potential to produce RA and that replication competent HSV-2 induces increased expression of the rate-limiting enzyme that converts retinaldehyde to RA.

### HSV-2 infection enhances HIV-1 replication in a RA-dependent manner

**Figure 6:**
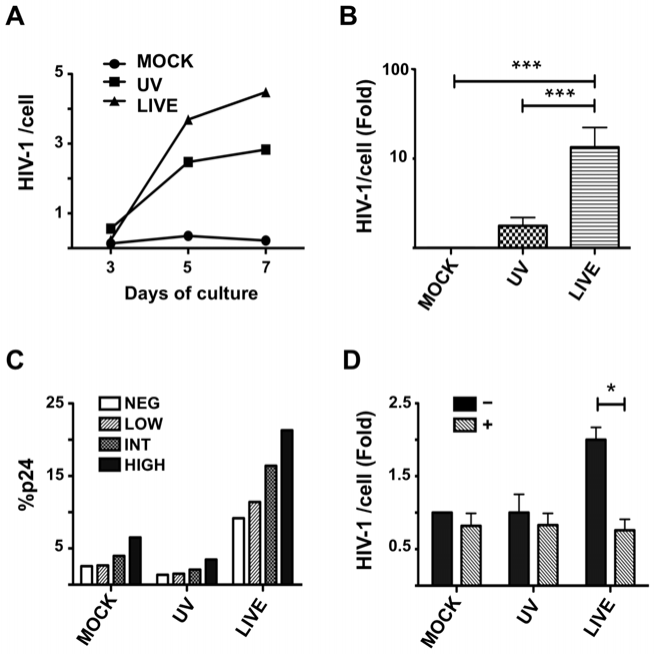
HSV-2 infection enhances HIV-1 replication in a RA-dependent manner. Mock-, UV-HSV-2-, or Live HSV-2-treated DCs were loaded with HIV-1 and co-cultured with autologous CD4^+^ T cells for 3 to 7 days (A) or 5 days (B–E). A) The kinetics of infection is shown for 1 of 3 similar experiments. B) The fold changes (mean ± SEM of 15 independent experiments) in HIV-1 DNA copies/cell for the 3 conditions after 5 days of co-culture are shown relative to the MOCK controls (set as 1). C) The percentage of p24^+^CD4^+^ T cells within each α_4_β_7_ subset was measured 5 days after co-culture with the differently treated DCs. 1 of 3 independent experiments is shown. D) The fold changes (mean ± SEM, 4 independent experiments) in the HIV-1 copies/cell in presence (+) or absence (−) of the RARα antagonist are shown relative to the mock-treated controls (set as 1). One experiment, in which the HIV-1 infection was about 100 fold higher in the live HSV-2 condition compared to the mock treated, was excluded. In the excluded experiment the RARα antagonist did not reverse the HSV-2-infected DC enhancement effect (reduced by 20%). (*p<0.05, **<0.01, ***p≤0.001).

Previous studies showed that RA profoundly affects HIV-1 replication *in-vitro* [13], [14], [15]. Having shown that HSV-2-infected DCs increase the percentage of α_4_β_7_^high^ T cells through an RA-dependent mechanism, we evaluated the impact of HSV-2 infection of immature moDCs on HIV-1 replication in DC-T cell mixtures. HSV-2-infected (versus UV-HSV-2- and mock-treated DCs) were pulsed with the CCR-5 tropic HIV-1 ADA-M, extensively washed and then mixed with autologous CD4^+^ T cells. HSV-2 infection of DCs significantly increased HIV-1 infection in CD4^+^ T cells in the DC-T cell co-cultures (Figure 6A–C). Although there was high variability in the level of HIV-1 infection and enhancement from donor to donor, the HSV-2-driven increase was consistent and in 1 out of 15 experiments reached a 100-fold increase compared to the mock condition. We found a modest increase in HIV-1 replication of UV-HSV-2-treated DC-T cell co-cultures compared with the mock condition, but this was not statistically significant when data from multiple donors was evaluated (Figure 6B). However, higher amounts of UV-HSV-2 induced higher HIV-1 replication (Figure S6 B), paralleling the increased CD69 expression seen in these cultures (Figure 4C and S4 B).

As previously suggested [21], [22], the α_4_β_7_^high^CD4^+^ T cells are highly susceptible to HIV-1 infection, as evidenced by p24 expression in the various T cell subsets (Figure 6C). Specifically, the percentage of p24^+^ cells in the α_4_β_7_^high^ subset was always higher than in the α_4_β_7_^int^ subset [1.6 fold, 95%CI: 1.1–2.1], than in the α_4_β_7_^low^ subset [4.8 fold, 95%CI: 1.5–8.2] and than in the α_4_β_7_^neg^ subset [2.8 fold, 95%CI: 2.4–3.2] (Figure 6C). In all experiments, the greater difference between the α_4_β_7_^high^ and the other subsets was found in the HSV-2-infected DC-containing cultures.

Since T cells can be infected with HSV-2 (Figure S5 A and [36]), we examined the effect of HSV-2 infection of CD4^+^ T cells on HIV-1 replication (in absence of DCs). We found a small increase in HIV-1 replication in the CD4^+^ T cells treated with replication competent HSV-2 compared to the mock condition similar to the increase in the UV control (Figure S7 A), coincident with the increased CD69 expression within these cultures (Figure S5 C).

Next we tested the effect of the RARα antagonist on the HIV-1 infection in the DC-T cell cultures. Inclusion of the RARα antagonist blocked the increased HIV-1 replication seen in the presence of HSV-2-infected DCs, but had low or no impact on the HIV-1 replication in the mock- and UV-HSV-2-treated DC-T cell mixtures (Figure 6D). With the exception of the experiment exhibiting 100-fold increase in HIV-1 replication, the RARα antagonist was able to inhibit infection to a level similar to that of the mock DC-T co-cultures (Figure 6D). The RARα antagonist had no effect on low-level HIV infection of CD4^+^ T cells cultured in the absence of DCs (Figure S7 B). These data confirm that HSV-2-infection of DCs augments HIV-1 replication in DC-T cell mixtures via an RA-dependent pathway.

## Discussion

HSV-2 enhances HIV-1 acquisition and transmission during symptomatic and asymptomatic stages of HSV-2 infection [2]. However, due to the lack of a suitable animal model for HSV-2 infection that closely relates to humans, the underling mechanism(s) that leads to enhanced risk of HIV-1 infection remains unknown. One potential explanation holds that increased presence and persistence of HSV-2-reactive CD4^+^ T cells facilitate HIV-1 transmission [3].

Herein, we provide *in-vivo* data collected in a novel non-human primate model of HSV-2 infection and we describe *in-vitro* experiments that add new insights into HSV-2/HIV-1 interplay. *In-vivo* we observed an increase in the percentages of α_4_β_7_^high^CD4^+^ T cells both locally and systemically a few days after rectal HSV-2 challenge. We show that CD11c^+^ DCs from peripheral blood are susceptible to HSV-2 infection *in-vitro* and that HSV-2 infection of immature moDCs amplifies the α_4_β_7_^high^ CD4^+^ T subset in autologous DC-T co-cultures. We show that HSV-2 infection increases ALDH1A1 expression in DCs, a phenomenon that enhances their potential to produce RA. The latter mediates the HSV-2-driven up-regulation of α_4_β_7_ in our DC-T co-cultures and has a plethora of immunomodulatory effects, including influencing HIV-1 replication [13], [15] Indeed, we found that blocking the RARα in T cells inhibits HIV-1 replication in HSV-2-infected DC-T cell cultures.

Localization, retention, function and survival of antigen-experienced T cells that infiltrate mucosal sites following pathogen invasion, are influenced by the expression of adhesion molecules, substantially modulated by microenvironmental factors [38]. Among such adhesion molecules, the integrin receptor α_4_β_7_ mediates lymphocyte migration to the gastrointestinal tract. However, recent findings indicate that STIs can modulate the expression and migration of α_4_β_7_^+^ lymphocytes also in other tissues, such as the endocervix of human females infected with *Clamydia trachomatis* [39], [40]. We developed a macaque HSV-2 rectal infection model and show that in macaques the mucosal site of HSV-2 infection, its draining LNs, and blood are enriched in α_4_β_7_^high^ T cells within 6 days of HSV-2 exposure. The increased percentages of α_4_β_7_^high^ T cells were not observed in animals treated with UV-HSV-2, suggesting that HSV-2 replication is important to this phenomenon.

Several factors could explain the enrichment in α_4_β_7_^high^ T cells at the site of HSV-2 infection. Among them is the ability of α_4_β_7_^high^ T cells to specifically target mucosal sites, a possible inflammation-driven induction of the α_4_β_7_ receptor MadCam [41], [42], and specific responses of CM T cells to inflammatory soluble factors. However, DCs are present and persist at the site of HSV-2 infection [3], they are critical to the immunological response to HSV-2 [43], [44], [45] and are able, in determinate circumstances, to induce α_4_β_7_ on T cells. Therefore, we explored the possibility of their contribution to the increased percentage of α_4_β_7_^high^ T cells in HSV-2-infected macaques.

HSV-2 is able to skew DC immunological responses [5], [6], [46]. While moDCs and langerhans cells are highly susceptible to HSV *in-vitro* [4], [5], [6], [7], plasmacytoid DCs, critical players in the innate response to HSV [47], seem to be resistant to infection [48]. CD11c^+^ myeloid DCs are important in antigen presentation and adaptive response to HSV [45]. Mimicking the mixed leukocyte populations potentially encountering HSV-2 *in-vivo* using blood, we confirmed that (macaque and human) myeloid CD11c^+^ DCs are the primary leukocyte target for HSV-2 infection *in-vitro*. Due to the variety of CD11c^+^ DC subsets implicated at different stages of the immune response [44] future studies will need to investigate the precise phenotype of the susceptible population, the differences between HSV-2-infected *in-vitro* generated moDCs, and infected CD11c^+^ DCs in their interaction with T cells. Since the primary goal of this study was to explore whether modulation of myeloid DC function by HSV-2 infection was involved in the enrichment of α_4_β_7_^high^ T cells observed *in-vivo*, we were able to use the established moDC-HSV-2 model to dissect this biology.

The effect of HSV-2 infection on moDCs has been extensively studied, typically using relatively large amounts of virus [4], [5], [6], [7]. Our work reveals that even a much smaller viral inoculum significantly influences DC biology. We confirmed that low dose HSV-2 infection caused a down-regulation of the maturation receptors HLA-DR, CD86 and CD54, as seen with higher HSV-2 doses [49], [50]. We also demonstrated a down-regulation of CD209, which would be expected in a maturing DC. The latter can be explained by the ability of HSV to bind this receptor [7], although it could be also the result of a skewed maturation process. We demonstrated that DCs infected with a low HSV-2 inoculum down-modulated CD69 expression on T cells, supporting an earlier report that HSV-infected moDCs inhibit T cell activation. Notably, we found that HSV-2-infected DCs up-regulate the expression of α_4_β_7_ and, by blocking the binding of RA to its receptors on the CD4^+^ T cells, we showed that RA was directly involved in the HSV-2 driven increase in α_4_β_7_ expression. RA impacts several immunological mechanisms, in particular it is known to induce a mucosal-type phenotype in DCs [51], playing an important role in inducing and sustaining the tolerogenic microenvironment of the gut [25].

We provide the first evidence that human immature moDCs express ALDH1A1 (and ALDH1A2), have the potential to convert serum retinol into RA, and that HSV-2 infection significantly increases this capability. This supports earlier work in mice showing that GM-CSF and IL-4 induce ALDH1A2 expression in BM-DCs [32]. The same study also reported that this gene was up-regulated by TLR ligands in DCs cultured with GM-CSF and IL-4 and matured with LPS. However, the up-regulation of ALDH1A1 expression by HSV-2 infection in human moDCs did not appear to be due a ligand effect of HSV-2 proteins or DNA (triggering through TLRs), since even 25-times more UV-HSV-2 was unable to reproduce these responses. Additional studies are needed in order to ascertain whether other TLR ligands or other pathogens can stimulate human DCs (like HSV-2 infection) to up-regulate ALDH1A1 expression and subsequently increase α_4_β_7_ expression on CD4^+^ T cells. The specific mechanism through which HSV-2 infection increases ALDH1A1 expression in moDCs was not a major focus of this work and might be a direct effect of newly synthesized HSV-2 components and/or an indirect effect of CCs/CKs secreted by DCs in response to HSV-2 replication. Given the potentially important role that RA holds in immune responses to pathogens, this subject is worthy of further research. That HSV-2 is able to mediate the up-regulation of an enzyme that serves as a key metabolic checkpoint in the conversion of retinol to RA is noteworthy, because RA has the capacity to modulate immune responses and replication of many pathogens including HIV-1 [15], [25], [52].

These studies also revealed that significantly elevated amounts of other soluble factors are released by HSV-2-infected moDCs. In particular, we detected a notable increase in IL-7, which is known to induce HIV-1 reactivation and replication in T cells [53], [54] and, as previously reported [55], of CXCL10 which is responsible of recruiting activated T cells, therefore contributing to viral replication in inflamed tissues [56]. All these factors could cooperate in enhancing HIV-1 infection. However, an RAR antagonist ablated the HSV-2-mediated enhancement of HIV-1 amplification, suggesting that RA is one of the major factors driving this biology.

HIV-1 infection of moDCs could also be affected by HSV-2 infection. Though, the apoptotic nature of the HSV-2 infection, suggests little contribution of HIV-1 replication in moDCs to the enhanced HIV-1 replication in the co-cultures.

We previously reported that α_4_β_7_^high^ T cells are the most susceptible HIV-1 target in T cells cultures supplemented with RA and that blocking α_4_β_7_ binding to HIV-1 inhibits HIV-1 replication [20], [21]. Herein, we show that the α_4_β_7_^high^ T cells also constitute the most susceptible HIV-1 target in the DC-T cell co-cultures and that this is independent of the effect of HSV-2 on the DCs. Therefore, being particularly susceptible to HIV seems an intrinsic characteristic of α_4_β_7_^high^CD4^+^ T and an expansion of this cell-subset likely has a greater impact than the expansion of less susceptible subsets, contributing to fuel infection.

**Figure 7:**
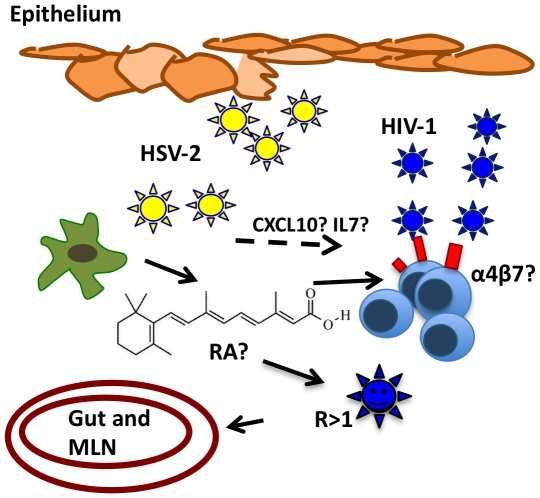
Potential mechanism of HSV-2-infected DCs enhancement of HIV-1 infection. Rapid replication of HSV-2 in epithelial cells could drive a low level HSV-2 infection in neighboring DCs, imprinting on them a “mucosal-like” phenotype and inducing the ability to release RA. RA can profoundly affect resident and/or recruited lymphocytes and expand the pool of α_4_β_7_^high^CD4^+^ T cells at the mucosal site of infection. These highly susceptible cells could contribute to increase HIV-1 replication rate and HIV-1 rapid access gut inductive sites, PPs and MLNs. Therefore, the RA-driven immunomodulatory effect and other HSV-2 driven changes – that need to be investigated - could partner to create an environment particularly susceptible to HIV-1 infection.

This work gives us new insights into HSV-2 modulation of the mucosal microenvironment. A low- level HSV-2 infection of immature myeloid DCs could play a role in increasing the susceptibility to HIV-1 by influencing its surroundings in a way favorable to HIV-1 infection. In Figure 7 we try to integrate our findings in a bigger picture with the new different actors that HSV-2 infected DCs add to the scene. Further studies will have to dissect how these mechanisms interplay *in-vivo*, the respective role of factors such as RA and α_4_β_7_ and their relative importance in transmission across the rectal and genital mucosa.

## Materials and Methods

### Ethics statement

Adult female Chinese rhesus macaques (*Macaca mulatta*) were housed and cared for in compliance with the regulations under the Animal Welfare Act, the Guide for the Care and Use of Laboratory Animals, at Tulane National Primate Research Center (TNPRC; Covington, LA). Animals were monitored continuously by veterinarians to ensure their welfare. Veterinarians at the TNPRC Division of Veterinary Medicine have established procedures to minimize pain and distress through several means. Monkeys were anesthetized with ketamine-HCl (10 mg/kg) or tiletamine/zolazepam (6 mg/kg) prior to all procedures. Preemptive and post procedural analgesia (buprenorphine 0.01 mg/kg) was required for procedures that would likely cause more than momentary pain or distress in humans undergoing the same procedures. The above listed anesthetics and analgesics were used to minimize pain or distress associated with this study in accordance with the recommendations of the Weatherall Report. Any sick animals were euthanized using methods consistent with recommendations of the American Veterinary Medical Association (AVMA) Panel on Euthanasia. All studies were approved by the Animal Care and Use Committee of the TNPRC (OLAW assurance #A4499-01) and in compliance with animal care procedures. TNPRC is accredited by the Association for Assessment and Accreditation of Laboratory Animal Care (AAALAC#000594).

### Cells and reagents

Immature moDCs were generated as previously described [6]. Briefly: Peripheral blood mononuclear cells (PBMCs) were isolated from heparinized human leukopacks (New York Blood Center, New York, NY) using Ficoll-Hypaque density gradient centrifugation (Amersham Pharmacia Biotech, Uppsala, Sweden). CD14^+^ monocytes were isolated using the human CD14 magnetic cell sorting (MACS) system (Miltenyi Biotec, Auburn, CA) and moDCs generated by culturing these cells in 100 U/mL recombinant human interleukin-4 (IL-4) (R&D Systems, Minneapolis, MN) and 1000 U/mL recombinant human granulocyte-macrophage colony-stimulating factor (GM-CSF) (Berlex Laboratories, Montville, NJ). After 5 days, immature moDCs were collected, an aliquot taken for flow cytometric analysis (BD FACSCAlibur) of DC phenotype and maturation [FITC anti–HLA-DR, APC anti-CD25, PE anti-CD80, PE anti-CD83, anti-CD86, PE anti-CD3 (see later for clones)]. moDC purity was greater than 98%. Cells were negative for CD80 and CD25; less than 1% of moDCs expressed CD83. The remainder of the cells was used for HSV-2 infections. moDCs were cultured in R1 [RPMI 1640 (Cellgro; Fisher Scientific, Springfield, NJ) containing 2 mM L-glutamine (GIBCO Life Technologies, Grand Island, NY), 10 mM HEPES GIBCO Life Technologies), 50 µM 2-mercaptoethanol (Sigma Chemical, St Louis, MO), penicillin (100 U/mL)/streptomycin (100 µg/mL) (GIBCO Life Technologies), and 1% heparinized human plasma]. Autologous CD14^−^ cells were cultured 5 days in R10 [RPMI 1640 with 2 mM L-glutamine, 10 mM HEPES, 50 µM 2-mercaptoethanol, penicillin 100 U/mL/streptomycin and 10% of FBS] supplemented with 1 U/ml of IL2 (Preclinical Repository, National Cancer Institute at Frederick, NCI-Frederick, MD) at 20×10^6^ cells/ml. The day before starting the DC-T culture, day 5, CD4^+^ T cells were isolated using the human CD4 positive MACS system (Miltenyi). CD4^+^ T cells were cultured overnight in R5 [RPMI 1640 with 2 mM L-glutamine, 10 mM HEPES, 50 µM 2-mercaptoethanol, penicillin 100 U/mL/streptomycin and 5% of human AB serum (Sigma-Aldrich)] supplemented with 1 U/ml of IL2, at 10×10^6^ cells/ml.

### HSV-2 infections

Viral stocks were propagated in Vero cells (American Type Culture Collection [ATCC] Manassas, VA), titered by plaque formation on Vero cells, and aliquots stored at −80°C [57]. HSV-2 was inactivated by exposure to UV lamp for 6 h in 6 wells plates without lid. Inactivation was verified by plaque formation on Vero cells. Freshly isolated human and macaque PBMCs were resuspended in cold RPMI 1640 at 50×10^6^ cells/ml and exposed to 0.2 or 5 plaque forming units (pfu)/cell (1 MOI = 1 pfu/cell) of live or UV-inactivated HSV-2 or to the equivalent volume of medium in which the viral stocks were grown (mock) (Dulbecco modified Eagle medium [DMEM] 2% FBS) for 2 h at 37°C cells were extensively washed and cultured for 24 h in R10. 1 U/ml of IL2 was added to the PBMC cultures.

Immature moDCs were collected at day 5, resuspended in cold RPMI 1640 without FBS at 20×10^6^ cells/ml, exposed to live 2 (0.04, 0.2 or 1 pfu/cell) or UV-inactivated HSV-2 (0.2, 1 or 5 pfu/cell) or to DMEM (mock-treated) for 2 h at 37°C. Cells were extensively washed and cultured for 24 h in R1 (with 100 U/mL IL-4 and 1000 U/mL GM-CSF) at 1×10^6^ cells/ml in 6 wells plates. HSV-2 infection was confirmed at 4 and 24 h by flow cytometry (BD FACSCAlibur). Although not a perfect control, 5 pfu/cell of UV-HSV-2 (25 fold the live inoculum) was rationalized from the infection of Vero cells. These cells release a maximum amount of virus corresponding to 16 folds their inoculum after 24 h in culture.

### PBMC and moDC immunostaining

HSV-2-infected PBMCs were washed and resuspended in PBS with LIVE/DEAD Fixable Aqua (Invitrogen, Life Technologies) for 10 mins at 4°C. Cells were washed with PBS, resuspended in FACS wash buffer (PBS 5% BSA 0.1% Na Azide) and incubated for 30 mins at 4°C with: Pacific Blue anti-CD3 (clone SP34-2) and anti-CD14 (clone M5E2), Alexa700 anti-CD20 (clone 2H7), PCP-Cy5.5 anti-HLA-DR (clone L243), PeCy7 anti-CD11c (clone 3.9). Cells were washed with FACS wash and fixed/permeabilized with Fix/Perm and Wash/Perm buffers (BD Biosciences). Cells were stained with anti-HSV-2 ICP-8 monoclonal antibody (mAb) (IgG2a isotype; Virusys, North Berwick, ME) 15 mins at room temperature, washed and analyzed within 24 h with BD LSRII. The ICP-8 mAb was directly conjugated with Alexa647 (Zenon Antibody labeling kit, Invitrogen, Life Technologies). Data were analyzed with FlowJo software 8.8.6.

HSV-2-infected and mock-treated moDCs were washed and resuspended in PBS with LIVE/DEAD Fixable Aqua for 10 mins at 4°C. Cells were washed with PBS, resuspended in FACS wash buffer incubated 30 mins at 4°C with FITC anti–HLA-DR (clone L243), PE anti-CD25 (M-A251), PE anti-CD80 (clone L307.4), PE anti-CD86 (clone), PE anti-CD209 (clone DCN46), PE anti-CD40 (clone 5C3), PE anti-CD54 (clone HA58) PE anti-CD83 (clone HB15e) (all BD Biosciences). Cells were fixed and permeabilized with Fix/Perm and Wash/Perm buffers (BD Biosciences). Cells were stained with anti–HSV-2 ICP-8 mAb 15 mins at room temperature, washed and analyzed within 24 h with BD FACS Calibur or with BD LSRII, when stained with Aqua.

### DC-T cell assays

HSV-2-infected (0.2 MOI) versus UV-HSV-2- and mock-treated DCs were resuspended in PBS 1% BSA at 10×10^6^ cells/ml and exposed to 8×10^4^ TCID_50_/10^6^ cells of HIV-1 ADA-M (Lot: P4023. Sucrose gradient-purified was kindly provided by the AIDS Vaccine Program, SAIC-Frederick, NCI-Frederick) for 2 h at 37°C (versus no virus controls as indicated). 30 mins before the end of the 2 h, CD4^+^ T cells were resuspended at 6×10^6^ cells/ml in R5 supplemented with 1 U/ml of IL-2 and plated in 48 well plates. RAR antagonist Ro41-5253 (Enzo Life Sciences, Zandhoven Belgium) at the final concentration of 500 nM or the same quantity of dimethyl sulfoxide (DMSO) was added to the wells for 10 mins at room temperature. HIV-1-pulsed moDCs were washed 3 times and resuspended at 2×10^6^ cells/ml in R5 (1 U/ml IL2). Cells were mixed at a 1∶3 ratio in 48 well plates (0.5×10^6^ DC: 1.5×10^6^ T cells) and cultured at a final concentration of 4×10^6^ cells/ml. Control CD4^+^ T cells were cultured at 4×10^6^ cells/ml in R5 (1 U/ml IL2), exposed to live, UV HSV-2 or DMEM and/or co-exposed to 8×10^4^ TCID_50_/10^6^ cells of HIV-1 ADA-M (Lot: P4023) for up to 5 days. Viruses were added directly to the CD4^+^ T cells cultured in absence of moDCs and not washed out. After 3, 5 and 7 days pellets and supernatants from the HIV-infected DC-T cell and T cell only cultures were collected and stored at −80°C.

HIV-naive DC-T cell and T cell only cultures were stained with LIVE/DEAD Fixable Aqua (Invitrogen, Life Technologies), PerCP-Cy5.5 anti-CD4 (clone SP34-2), Pacific Blue anti-CD3 (clone L200), Alexa700 anti-CD69 (Clone FN50), FITC anti-CD45RA (clone HI100), APC anti-CD45RO (clone UCHL1), FITC anti-CD62L (clone SK11), PeCy7 anti-CCR7 (clone 3D12), PE anti-dimeric α4β7 (Act-1 Clone, NIH AIDS Research Reference and Reagent Program). Act-1 was directly conjugated using LYNX RPE antibody Conjugation KIT (AbD Serotech, Raleigh, NC) at 4°C for 30 mins. Cells were fixed in Cytofix buffer (BD Bioscience). At least 200000 events in the lymphocyte gate were acquired and analyzed using the BD LSRII Flow Cytometer and the FlowJo 8.8.6 software (Tree Star, Inc.).

### HIV- qPCR

DNA from DC-T cell pellets was extracted using DNeasy Blood & Tissue kit (Qiagen Inc, Valencia, CA). Quantitative PCR for HIV *gag* DNA and an estimation of cell numbers using albumin DNA copy numbers was performed using published primers and molecular probes [58]. DNA was quantified by qPCR with an ABI 7000 PCR machine (PerkinElmer Life and Analytical Sciences, Boston, MA) using 5 µl of DNA for 40 cycles and the ABI master mix (TaqMan Universal PCR Master Mix; Applied Biosystems).

### ALDH1A RT- qPCR

ALDH1A Primers are listed in Supplementary Table S2 and were generated using Primers3 [59]. They were designed to span at least 1 exon-exon junction and their specificity was verified by nucleotide blast. The GAPDH gene was used as positive assay control with primer sequences: FW GAAGGTGAAGGTCGGAGT, RW GAAGATGGTGATGGGATTTC. RNA extraction was carried out using RNeasy Mini Kit (Quiagen) and residual DNA was digested using the RNase-Free DNase Set (Quiagen). The reverse transcription (RT) was performed using RandomPrimers (Invitrogen), dNTPmix (BioRad) DTT (5 µM, Invitrogen) Superscript III RT and 5× First strand buffer (Invitrogen) and the MyCycler, Thermal Cycler (Bio-Rad, Laboratories, Inc., Hercules, CA) Cycling conditions: 25°C 5 mins, 50°C 45 mins, 70°C 15 mins. RNase H (Invitrogen) was used to remove RNA template. The PCR was performed using HotStarTaq Master Mix (Qiagen). Cycling conditions: 95°C 10 mins, 25×(94°C 30 sec, 60°C 30 sec, 72°C 30 sec), 72°C 10 mins. mRNA from CD14^−^ PBMCs was used as negative control for ALDH1A expression. The RT step for quantitative PCR (qPCR) was carried out using the SuperScript Vilo cDNA synthesis kit (Invitrogen). 100 ng of RNA was used in each reaction. Relative qPCR was performed using the SYBR Green PCR Master Mix (Applied Biosystems, Life Technologies). 1 µl of cDNA was used in each reaction. The 7000 Sequence Detection System cycler (Applied Biosystems, Life Technologies) was used for carrying out the reaction. Cycling conditions: 95°C 10 mins, 40×(95°C 15 sec, 60°C 1 min). Dissociation curves were generated to verify absence of unspecific amplification. Data were analyzed using the ABI Prism 7000 SDS Software (Applied Biosystems). The standard curve was generated using 2 fold dilutions of RNA extracted from HSV-2 infected moDCs. RT of standards was performed each time together with the samples to avoid variation in the RT efficiency. In the real-time experiments GAPDH was used as endogenous control for sample normalization [60]. Arbitrary units were used to determine fold increase compared to uninfected (mock) moDCs.

### Analysis of ALDH activity

ALDH activity in individual cells was estimated using ALDEFLUOR staining kits (StemCell Technologies, Vancouver, BritishColumbia, Canada), according to the manufacturer's protocol with modifications as previously described [32]. Briefly, cells were suspended at 10^6^ cells/ml in ALDEFLUOR assay buffer containing activated ALDEFLUOR substrate (150 nM) with or without the ALDH inhibitor diethylaminobenzaldehyde (DEAB) on ice. Cells were incubated for 45 mins at 37°C, washed and stained with LIVE/DEAD fixable aqua (Invitrogen) for 30 mins on ice in ALDEFLUOR assay buffer. They were washed again and stained with APC anti-HLA-DR mAbs or isotype control for 30 mins on ice. ALDEFLUOR reactive cells were detected using BD LSRII flow cytometer with 488-nm blue laser and standard FITC 530/30 nm bandpass filter.

### Cytokine and Chemokine analysis

Stored cell culture supernatants were thawed and examined for CK/CC concentrations using the human cytokine 25-plex (Invitrogen) on the Luminex 200 (Luminex Corp. Austin, Texas). The kit measures IL-1β, IL-1RA, IL-2, IL-2R, IL-4, IL-5, IL-6, IL-7, CXCL8, IL-10, IL-12p40, IL-13, IL-15, IL-17, IFNα, IFNγ, TNFα, GM-CSF, CCL2, CCL3, CCL4, CXCL9, CCL11, CCL5. The StartStation software was used to analyze the data.

### Animals HSV-2 challenge

Animals were challenged intra-rectally with 2×10^8^ pfu of replication competent or UV-inactivated HSV-2 in 1 ml of serum-free RPMI 1640. Rectal swabs were collected 4 days and 1 day before HSV-2 challenge and 3 days and 6 days post challenge. Swabs were drained of fluid and then discarded. The remaining samples were centrifuged at 3500 rpm for 10 mins. Total fluids or aliquots of supernatants were stored at −80°C. Serum for SHIV-RT RNA levels was collected prior to challenge and at time of necropsy. Plasma was separated from whole blood by centrifugation at 2000 rpm for 10 mins, clarified at 2000 rpm for 10 mins and stored at −80°C in 1 ml aliquots. SIV *gag* RNA was measured by quantitative RT-PCR assay [61].

### HSV-2 nested PCR

HSV-2 DNA shedding was determined by measuring the presence of the HSV-2 gD gene (which encodes the viral entry receptor glycoprotein D) using a nested PCR. gD primers used: Ext FW AAGCGTGTTTACCACATTCAGCCG, RV TGTGTGATCTCCGTCCAGTCGTTT, Nested: FW TACTACGCAGTGCTGGAACG, RV CGATGGTCAGGTTGTACGTG. This assay was able to reproducibly detect HSV-2 gD DNA signals from 0.5 infected cells (single replicates) or 0.0005 infected cells (at least 2 positives in 6 replicates) [6], DNA was extracted from 0.3 ml aliquots of the total fluids using DNeasy Blood & Tissue kit (Qiagen). GAPDH primers and cycling conditions were performed as described above in the methods for the ALDH1A PCR.

### Animal cell isolation and flow cytometry

PBMCs were isolated from EDTA blood using Ficoll-Hypaque density gradient centrifugation. Axillary, inguinal, iliac and MLNs, as well as rectal tissues were obtained at necropsy. Fat tissue was removed from the LNs with a scalpel, LNs were cut in small pieces and passed through a 70 µm nylon cell strainer (BD-Falcon, Franklin Lakes, NJ). Cells were washed twice with RPMI and resuspended in FACS staining buffer. After removal of fat tissue and blood vessels, from the rectal mucosa was cut in small pieces and incubated in HBSS without Ca^2+^ and Mg^2+^ with 200 µg/ml gentamycin (Gibco, Life Sciences), 7.5 mg DTT (Sigma-Aldrich) and a final concentration of 1.7 mM of EDTA for 45 mins at room temperature on a shaking platform. HBSS was discarded and the remaining tissue washed in HBSS with Ca^2+^ and Mg^2+^. Tissues were incubated in R10 with 1 mg/ml hyaluronidase (Sigma-Aldrich) 0.5 mg/ml Collagenase II (Sigma-Aldrich) 1 mg/ml DNAse I (Roche) for 4 h at 37°C or until tissue was completely digested. The cell suspension was passed through a 70 µm nylon cell strainer, cells were washed twice with PBS and resuspended in FACS wash buffer. Cells were stained with PerCP-Cy5.5 anti-CD4 (clone SP34-2), Pacific Blue anti-CD3 (clone L200), Alexa700 anti-CD69 (Clone FN50), FITC anti-CD95 (clone DX2), APC anti-CD28 (clone 28.2), PE anti-dimeric α4β7 (Act-1 Clone) and PE-Cy7 anti-CCR7 (clone 3D12). At least 200000 events were acquired in the lymphocyte gate. Samples were analyzed using the BD LSRII Flow Cytometer.

### Statistics

Wilcoxon signed-rank and Mann-Whitney non-parametric tests were used to compare variables between groups (mock versus live HSV-2, UV-HSV-2 versus live HSV-2 and mock versus UV-HSV-2). Wilcoxon signed-rank and one sample t test with 1 as hypothetical value were both used to analyze results expressed as fold increase. A two-tailed P value α<0.05 was considered significant. Analysis was performed using Prism (GraphPad Software, Inc) version 5a.

### Accession numbers

NCBI Reference Sequences:

mRNA ALDH1A1: NM_000689.3 mRNA ALDH1A2: NM_170696.1 mRNA ALDH1A3: NM_000693.2

Swiss-Prot: ICP-8 P11870.2

## Supporting Information

Figure S1 **Gating strategy for α_4_β_7_^+^CD4^+^ T cells in rectal mucosa.** Cells isolated from rectal mucosa of clinically stable SHIV-RT-infected macaques were stained with the LIVE/DEAD discriminator fixable Aqua and mAbs against CD3, CD4, CD95 and α4β7. The respective gates are indicated in each panel.
(TIF)

Figure S2 **moDCs infected with 0.2 MOI of live HSV-2 are viable 24 h post infection.** A) moDCs were infected with 0.04, 0.2, 1 MOI of HSV-2 or treated with HSV-2 growing media (MOCK) and 24 h later stained with Annexin/PI. The percentages of early apoptotic cells (Annexin V^+^ PI^−^) and late apoptotic cells (Annexin V^+^ PI^+^) are shown for 1 of 2 independent experiments. B) 24 h HSV-2-infected (0.2 MOI), UV-HSV-2 or mock-treated moDCs were stained with the LIVE/DEAD BD fixable Aqua dye. The percentages of Aqua-low viable cells for 1 of at least 5 independent experiments are shown.
(TIF)

Figure S3 **Phenotype of the α4β7^high^CD4^+^ T cells in the DC-T cell co-cultures.** CD4^+^ T cells were co-cultured with HSV-2-infected DCs for 5 days. α4β7^high^ T cells are CD45RO^+^ (left). α4β7^high^CD45RO^+^ T cells are CD62L^+^CCR7^+^ (right). Plots are representative of more than 15 independent experiments.
(TIF)

Figure S4 **moDCs treated with a high dose of UV-HSV-2 do not induce α_4_β_7_ up-regulation on T cells.** Mock-, UV-HSV-2- (0.2 MOI and 5 MOI), or Live HSV-2- (0.2 MOI) treated DCs were mixed with autologous CD4^+^ T cells and cultured for 5 days. The fold changes (mean ± SEM, n = 11 for the mock and 0.2 MOI of UV or live HSV-2 conditions and n = 3 with 5 MOI of UV-HSV-2 condition) in the percentage of α_4_β_7_^high^CD3^+^CD4^+^ T cells (A) and of CD69^+^ CD3^+^CD4^+^ T cells (B) are shown. (*p<0.05, **<0.01, ***p≤0.001).
(TIF)

Figure S5 **Exposure of CD4^+^ T cells to live HSV-2 does not induce up-regulation of α_4_β_7_.** A-C) CD4^+^ T cells were exposed to 0.2 and 5 MOI of live HSV-2, UV-HSV-2 (0.2 MOI) or HSV-2 growing media (MOCK) for 5 days. The fold changes (mean ± SEM; 4 independent experiments) in the percentage of ICP-8^+^ (A), of α_4_β_7_^+^ in the low, intermediate and high subsets (B) and of CD69^+^ (C) cells are shown for each treatment group. In panels B and C, there was no difference in the percentages of α_4_β_7_^+^ and CD69^+^ cells in cultures exposed to 0.2 versus 5 MOI and so the data have been combined (B–C; total of 8 independent experiments) (*p<0.05).
(TIF)

Figure S6 **moDCs treated with high dose UV-HSV-2: effect on ALDH1A1 expression and HIV-1 replication.** A) The fold changes (mean ± SEM, n = 13 for the mock, 0.2 MOI of UV and Live HSV-2 conditions and n = 6 for the 5 MOI of UV-HSV-2 condition) in ALDH1A1 mRNA of LIVE and UV-HSV-2 treated moDCs (24 h post treatment) are shown relative to mock moDCs (set as 1). B) The fold changes (mean ± SEM, n = 15 the mock, 0.2 MOI of UV and Live HSV-2 conditions and n = 8 for the 5 MOI of UV-HSV-2 condition) in HIV-1 DNA copies/cell after 5 days of co-culture are shown relative to the MOCK controls (set as 1). (*p<0.05, **<0.01, ***p≤0.001).
(TIF)

Figure S7 **Exposure of CD4^+^ T cells to live HSV-2 does not significantly increase HIV-1 replication.** The fold changes (mean ± SEM, 10 independent experiments) in HIV-1 copies/cell in CD4^+^ T cells co-exposed to HIV-1 and 0.2 or 5 MOI of live, UV-HSV-2 (0.2 MOI) or HSV-2 growing media (MOCK) for 5 days. (B) The fold changes (mean ± SEM, 4 independent experiments) in the HIV-1 copies/cell in presence (+) or absence (−) of the RARα antagonist are shown relative to the mock-treated controls (set as 1).
(TIF)

Table S1 **Summary of CD4 counts, SIV viral loads and HSV-2 status.** CD4 counts before (BL) and after (D6 p.i.) HSV-2 challenge. SHIV-gag RNA levels in serum before (BL) and after (D6 p.i.) HSV-2 challenge. HSV-2 DNA detected (+) or not (−) before (BL) and 3 and 6 days (D3 and D6 p.i.) after HSV-2 challenge (LIVE) or treatment with UV inactivated HSV-2 (UV).
(DOC)

Table S2 **ALDH1A Primers.** Primer sequences used for RT-qPCR.
(DOC)
