# Supplementary figures and images for: HSV-2 Infection of Dendritic Cells Amplifies a Highly Susceptible HIV-1 Cell Target
Source: PLoS Pathog. 2011 Jun 30;7(6):e1002109. doi: 10.1371/journal.ppat.1002109 (PMC3128120; doi:10.1371/journal.ppat.1002109)

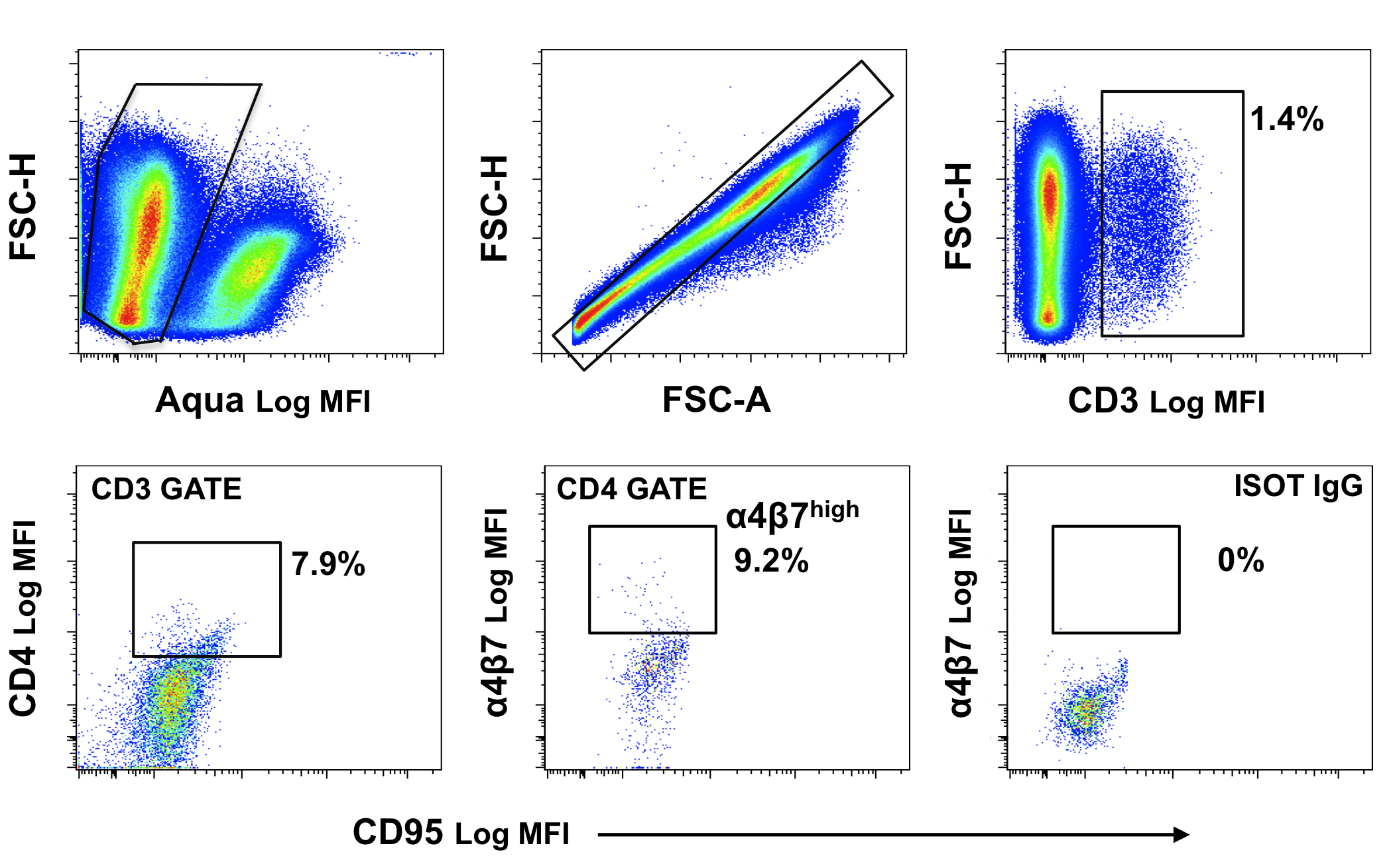

Supplement: Figure S1 — Gating strategy for α4β7+CD4+ T cells in rectal mucosa. Cells isolated from rectal mucosa of clinically stable SHIV-RT-infected macaques were stained with the LIVE/DEAD discriminator fixable Aqua and mAbs against CD3, CD4, CD95 and α4β7. The respective gates are indicated in each panel. (TIF) [file ppat.1002109.s001.tif]

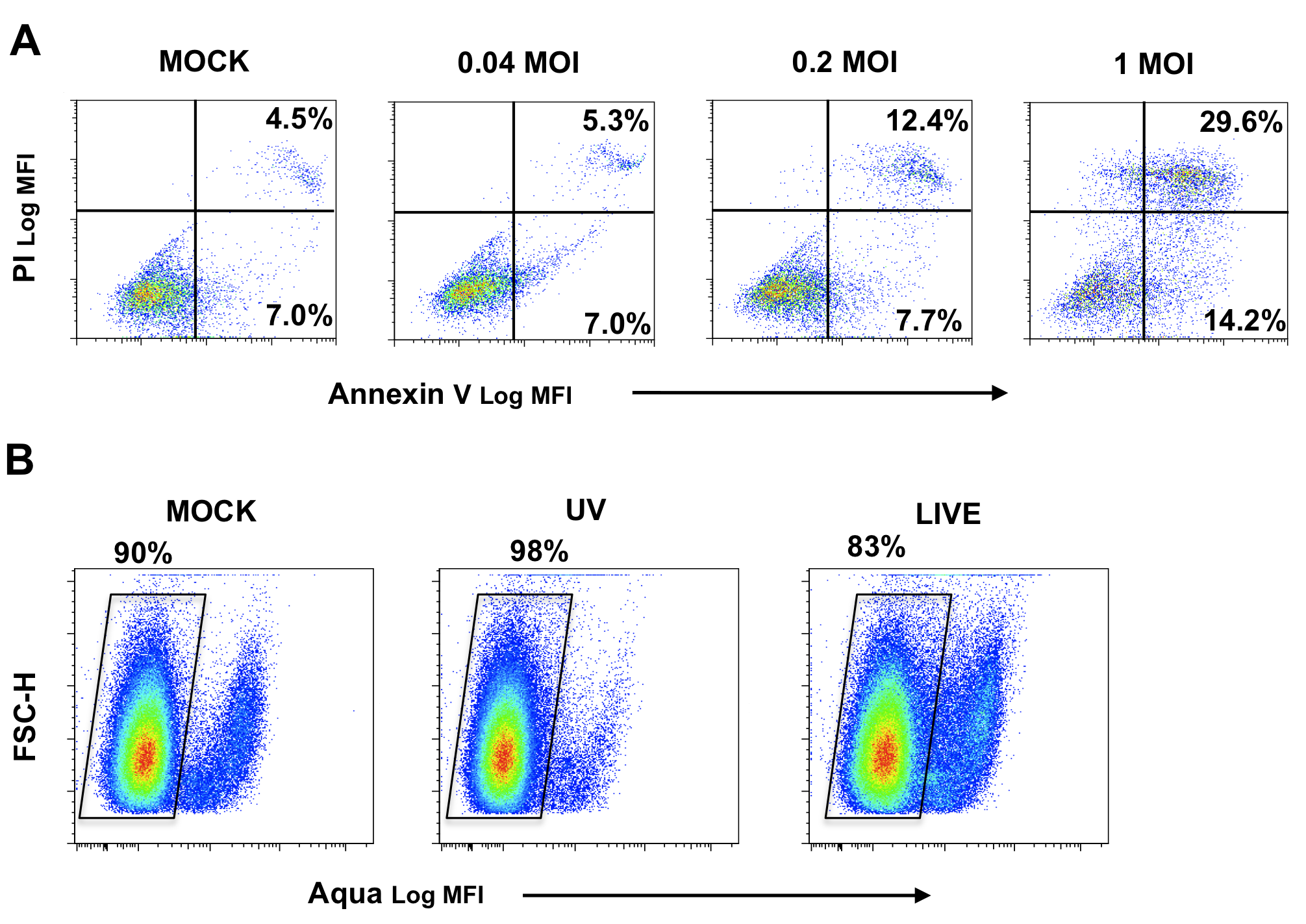

Supplement: Figure S2 — moDCs infected with 0.2 MOI of live HSV-2 are viable 24 h post infection. A) moDCs were infected with 0.04, 0.2, 1 MOI of HSV-2 or treated with HSV-2 growing media (MOCK) and 24 h later stained with Annexin/PI. The percentages of early apoptotic cells (Annexin V+ PI−) and late apoptotic cells (Annexin V+ PI+) are shown for 1 of 2 independent experiments. B) 24 h HSV-2-infected (0.2 MOI), UV-HSV-2 or mock-treated moDCs were stained with the LIVE/DEAD BD fixable Aqua dye. The percentages of Aqua-low viable cells for 1 of at least 5 independent experiments are shown. (TIF) [file ppat.1002109.s002.tif]

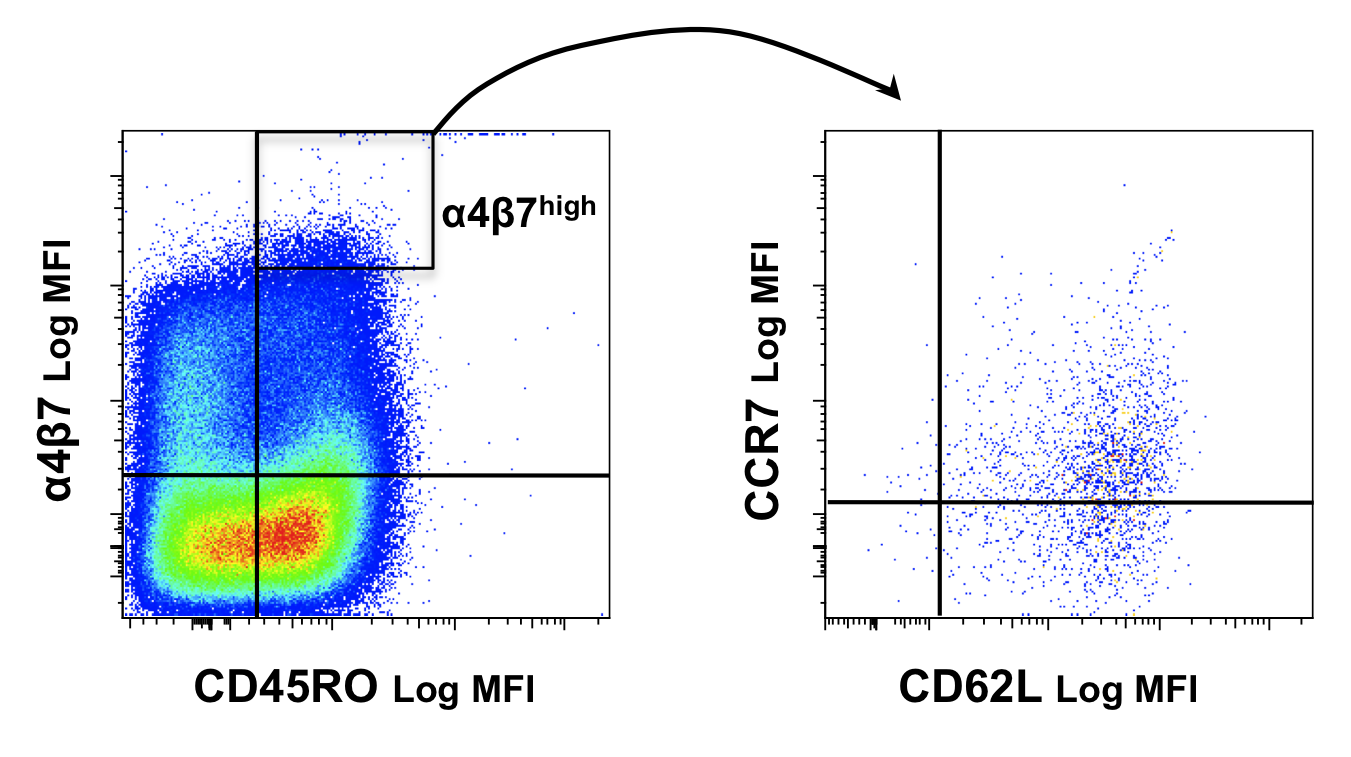

Supplement: Figure S3 — Phenotype of the α4β7highCD4+ T cells in the DC-T cell co-cultures. CD4+ T cells were co-cultured with HSV-2-infected DCs for 5 days. α4β7high T cells are CD45RO+ (left). α4β7highCD45RO+ T cells are CD62L+CCR7+ (right). Plots are representative of more than 15 independent experiments. (TIF) [file ppat.1002109.s003.tif]

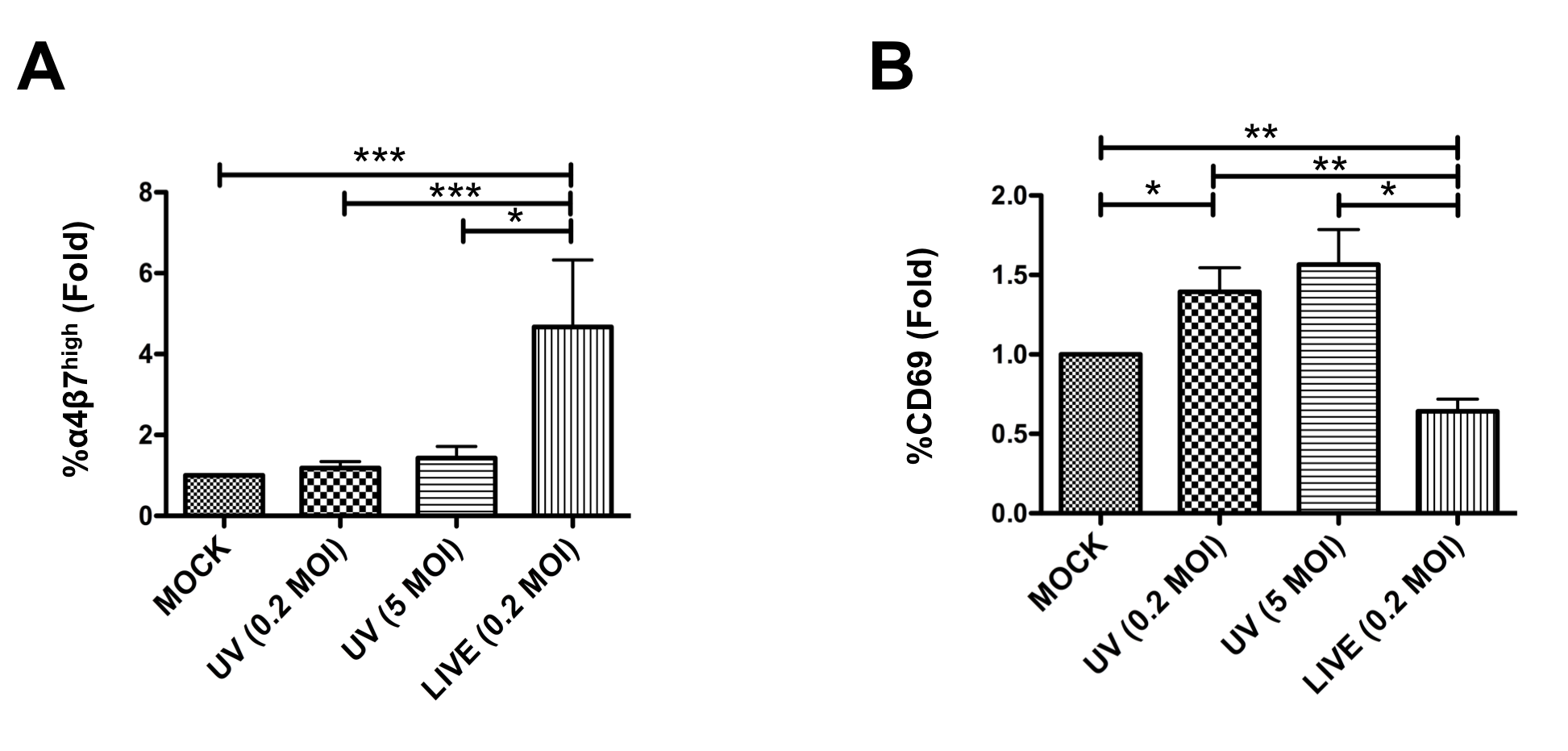

Supplement: Figure S4 — moDCs treated with a high dose of UV-HSV-2 do not induce α4β7 up-regulation on T cells. Mock-, UV-HSV-2- (0.2 MOI and 5 MOI), or Live HSV-2- (0.2 MOI) treated DCs were mixed with autologous CD4+ T cells and cultured for 5 days. The fold changes (mean ± SEM, n = 11 for the mock and 0.2 MOI of UV or live HSV-2 conditions and n = 3 with 5 MOI of UV-HSV-2 condition) in the percentage of α4β7 highCD3+CD4+ T cells (A) and of CD69+ CD3+CD4+ T cells (B) are shown. (*p<0.05, **<0.01, ***p≤0.001). (TIF) [file ppat.1002109.s004.tif]

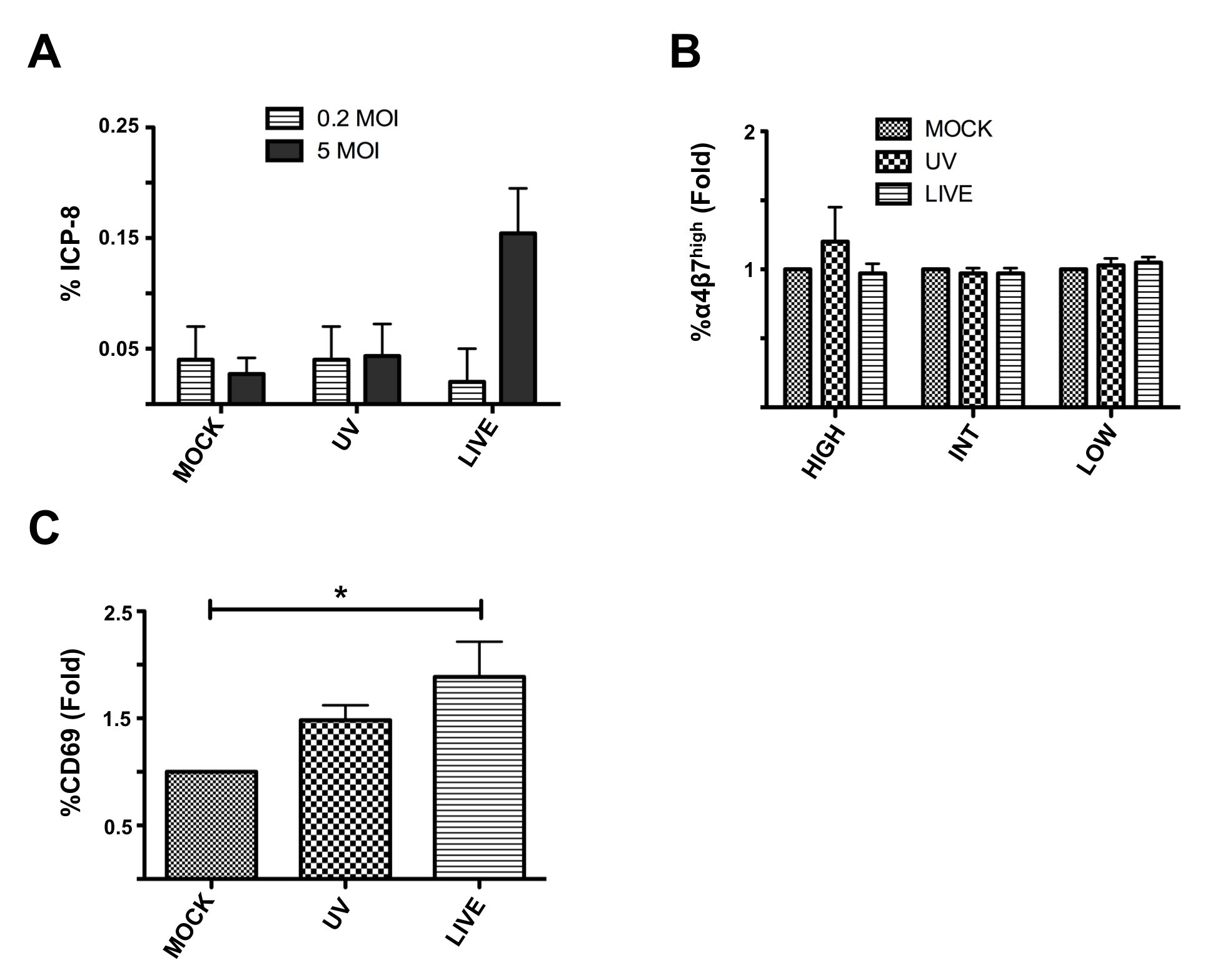

Supplement: Figure S5 — Exposure of CD4+ T cells to live HSV-2 does not induce up-regulation of α4β7. A-C) CD4+ T cells were exposed to 0.2 and 5 MOI of live HSV-2, UV-HSV-2 (0.2 MOI) or HSV-2 growing media (MOCK) for 5 days. The fold changes (mean ± SEM; 4 independent experiments) in the percentage of ICP-8+ (A), of α4β7 + in the low, intermediate and high subsets (B) and of CD69+ (C) cells are shown for each treatment group. In panels B and C, there was no difference in the percentages of α4β7 + and CD69+ cells in cultures exposed to 0.2 versus 5 MOI and so the data have been combined (B–C; total of 8 independent experiments) (*p<0.05). (TIF) [file ppat.1002109.s005.tif]

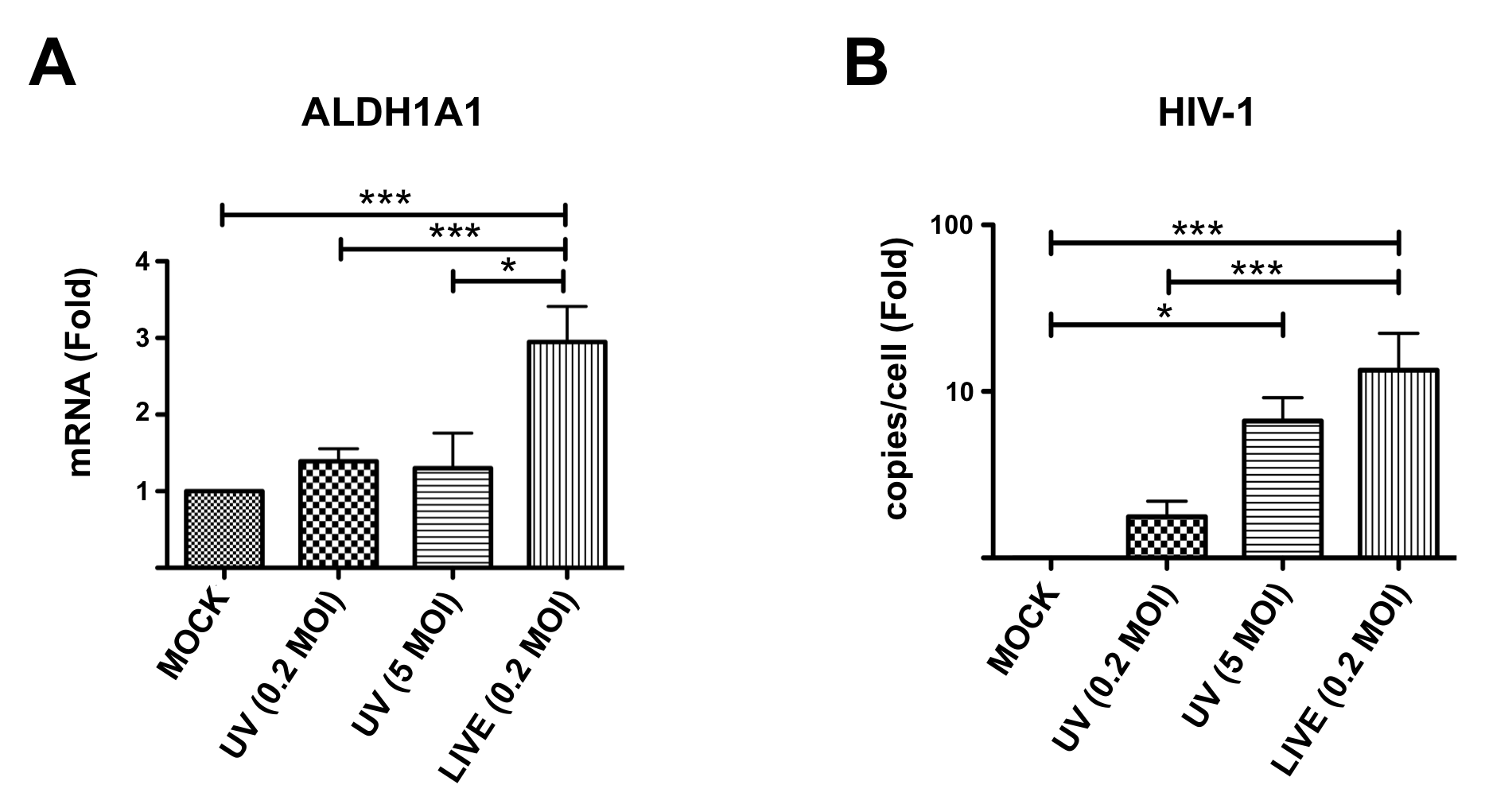

Supplement: Figure S6 — moDCs treated with high dose UV-HSV-2: effect on ALDH1A1 expression and HIV-1 replication. A) The fold changes (mean ± SEM, n = 13 for the mock, 0.2 MOI of UV and Live HSV-2 conditions and n = 6 for the 5 MOI of UV-HSV-2 condition) in ALDH1A1 mRNA of LIVE and UV-HSV-2 treated moDCs (24 h post treatment) are shown relative to mock moDCs (set as 1). B) The fold changes (mean ± SEM, n = 15 the mock, 0.2 MOI of UV and Live HSV-2 conditions and n = 8 for the 5 MOI of UV-HSV-2 condition) in HIV-1 DNA copies/cell after 5 days of co-culture are shown relative to the MOCK controls (set as 1). (*p<0.05, **<0.01, ***p≤0.001). (TIF) [file ppat.1002109.s006.tif]

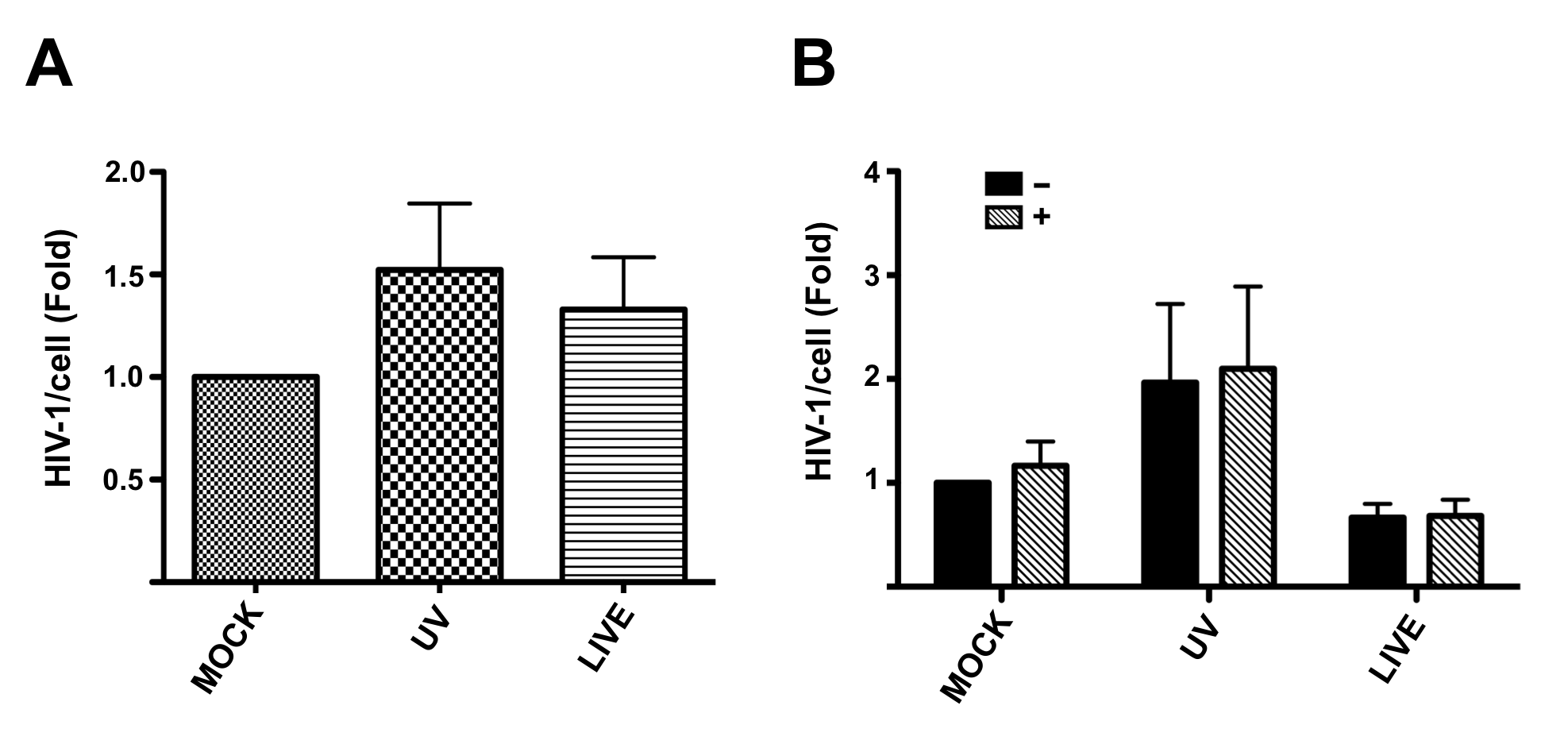

Supplement: Figure S7 — Exposure of CD4+ T cells to live HSV-2 does not significantly increase HIV-1 replication. The fold changes (mean ± SEM, 10 independent experiments) in HIV-1 copies/cell in CD4+ T cells co-exposed to HIV-1 and 0.2 or 5 MOI of live, UV-HSV-2 (0.2 MOI) or HSV-2 growing media (MOCK) for 5 days. (B) The fold changes (mean ± SEM, 4 independent experiments) in the HIV-1 copies/cell in presence (+) or absence (−) of the RARα antagonist are shown relative to the mock-treated controls (set as 1). (TIF) [file ppat.1002109.s007.tif]
