# Supplementary material for: HSV-2 Infection of Dendritic Cells Amplifies a Highly Susceptible HIV-1 Cell Target
Source: PLoS Pathog. 2011 Jun 30;7(6):e1002109. doi: 10.1371/journal.ppat.1002109 (PMC3128120; doi:10.1371/journal.ppat.1002109)
Supplement: Table S1 — Summary of CD4 counts, SIV viral loads and HSV-2 status. CD4 counts before (BL) and after (D6 p.i.) HSV-2 challenge. SHIV-gag RNA levels in serum before (BL) and after (D6 p.i.) HSV-2 challenge. HSV-2 DNA detected (+) or not (−) before (BL) and 3 and 6 days (D3 and D6 p.i.) after HSV-2 challenge (LIVE) or treatment with UV inactivated HSV-2 (UV). (DOC) [file ppat.1002109.s008.doc]

|  | **CD4 count** | | **Viral Load (x 10^3)** | | **Treatment** | **HSV-2 PCR** | | |
| --- | --- | --- | --- | --- | --- | --- | --- | --- |
| **Animal** | **BL**^a^ | **D6 p.i.** | **BL**^a^ | **D6 p.i.** | **HSV-2** | **BL**^a^ | **D3** | **D6** |
| **IE87** | 571 | 643 | 31 | 160 | LIVE | **-** | **+** | **+** |
| **GJ44** | 1344 | 1414 | < 0.03 | < 0.03 | LIVE | **-** | **+** | **+** |
| **GF31** | N/A | 1229 | N/A | N/A | LIVE | **-** | **+** | **+** |
| **HL66** | 480 | 348 | 64 | 57 | LIVE | **-** | **+** | **+** |
| **IE86** | 683 | 1032 | 1100 | 630 | LIVE | **-** | **+** | **+** |
| **IE82** | 772 | 516 | 340 | 160 | LIVE | **-** | **-** | N/A |
| **IE85** | 296 | 378 | 54 | 44 | LIVE | **-** | **+** | **+** |
| **HL47** | 886 | 1193 | .23 | .79 | LIVE | **-** | **+** | **+** |
| **HL49** | N/A | N/A | N/A | N/A | LIVE | - | **+** | **+** |
| **HL57** | N/A | N/A | N/A | N/A | LIVE | - | N/A | - |
| **GK05** | N/A | 189 | N/A | N/A | LIVE | - | **+** | **+** |
| **GT67** | 458 | 484 | < 0.03 | 3.7 | UV | **-** | **-** | **-** |
| **GT57** | 792 | 867 | < 0.03 | < 0.03 | UV | **-** | **-** | **-** |
| **IE78** | 847 | 700 | 33 | 50 | UV | **-** | **-** | N/A |
| **IE80** | 733 | 298 | 590 | 1200 | UV | **-** | **-** | **-** |
| **HL62** | N/A | 100 | N/A | N/A | UV | **-** | **-** | **-** |
| **HL61** | N/A | 510 | N/A | N/A | UV | **-** | **-** | **-** |
| **IE83** | 481 | 729 | 17 | 3.8 | UV | **-** | **-** | **-** |
| **GT65** | N/A | N/A | N/A | N/A | UV | **-** | **-** | **-** |

^a^ BL= 24h before challenge ^a^ N/A= not acquired
