# Supplementary material for: HSV-2 Infection of Dendritic Cells Amplifies a Highly Susceptible HIV-1 Cell Target
Source: PLoS Pathog. 2011 Jun 30;7(6):e1002109. doi: 10.1371/journal.ppat.1002109 (PMC3128120; doi:10.1371/journal.ppat.1002109)
Supplement: Table S2 — ALDH1A Primers. Primer sequences used for RT-qPCR. (DOC) [file ppat.1002109.s009.doc]

| **ALDH1A1**  NM_000689.3 | **Sequence (5'->3')** | **Start-stop** | **Tm** | **GC%** | **Exon junction** |
| --- | --- | --- | --- | --- | --- |
| FW | GCCATAACAATCTCCTCTGCTCTGC | 1371-1395 | 57.7 | 52 | n/a |
| RV | ACTCTCCCAGTTCTCTTCCATTTCCA | 1494-1469 | 57.21 | 46.15 | 1486/1487 |
| **ALDH1A2**  NM_170696.1 |  |  |  |  |  |
| FW | CCCTCATCAAGGAGGTTGGAAAGC | 729-752 | 57.45 | 54.17 | 742/743 |
| RV | GGTGGGCCTGCTCCACAGC | 872-854 | 59.96 | 73.68 | n/a |
| **ALDH1A3**  NM_000693.2 |  |  |  |  |  |
| FW | TGTGCGGACGCTGACTTGGAC | 972-992 | 59.66 | 61.90 | 988/1172 |
| RV | CTGACAAACTCAGAGTAGACCTGCTC | 1099-1074 | 56.77 | 50 | n/a |
